# Supplementary material for: Giant mid-IR resonant coupling to molecular vibrations in sub-nm gaps of plasmonic multilayer metafilms
Source: Light Sci Appl. 2022 Sep 23;11:281. doi: 10.1038/s41377-022-00943-0 (PMC9508334; doi:10.1038/s41377-022-00943-0)
Supplement: Supplementary file 1 — Supplementary Information [file 41377_2022_943_MOESM1_ESM.pdf]

Supplementary Information

Giant mid-IR resonant coupling to molecular vibrations in sub-nm gaps of plasmonic multilayer metafilms

Rakesh Arul, David Benjamin-Grys, Rohit Chikkaraddy, Niclas S Mueller, Angelos Xomalis, Ermanno Miele, Tijmen G Euser, and Jeremy J Baumberg*

Index to contents of Supplementary Information:

Section S1: Structural characterization of disordered AuNP aggregate films
Section S2: Optical properties of films aggregated with HCl, NaCl, and CB[5]
Section S3: Coupled-oscillator effective metamaterial model
Section S4: FDTD simulations of ordered AuNP superlattices and near fields
Section S5: Assignment of CB[5] infrared and Raman modes
Section S6: Xray photoelectron spectroscopy (XPS) of the monolayer CB[5] aggregated AuNP films
Section S7: SERS and SEIRA enhancement factor estimation
Section S8: Modified coupled-oscillator Fano resonance fitting
Section S9: Radiative decay rate of CB[5] within cavity, Purcell factor, and mode volumes
Section S10: SEIRA spectra of decane-1-thiol monolayers on multilayer films
Section S11: Multi-particle Mie-scattering simulations of disorder in 2D platelets of AuNPs
Section S12: Repeatability of fabrication of NPnML films
Section S13: Flow sensing within NPnML films and reusability studies

Section S1: Structural characterization of disordered AuNP aggregate films

The SEM images (Figure S1a) of the nanoparticles were analysed using the circular Hough transform, which was implemented in the imfindcircles function in Matlab R2019a, to extract the nanoparticle diameters (Figure S2) and centers (Figure S1b). The pair correlation function of the SEM images from the monolayer and bilayer NP aggregates on a mirror show that the particle-to-particle correlations do not extend far past the next-nearest neighbours (Figure S1c), and that the predominant coordination number is 5-6 (Figure S1d), which suggests disordered close-packed structures. AFM images of the multilayer regions (Figure S3) support the estimation of the coordination number from the SEM images. The surface packing density estimated from Figure S1b is 65.6%.

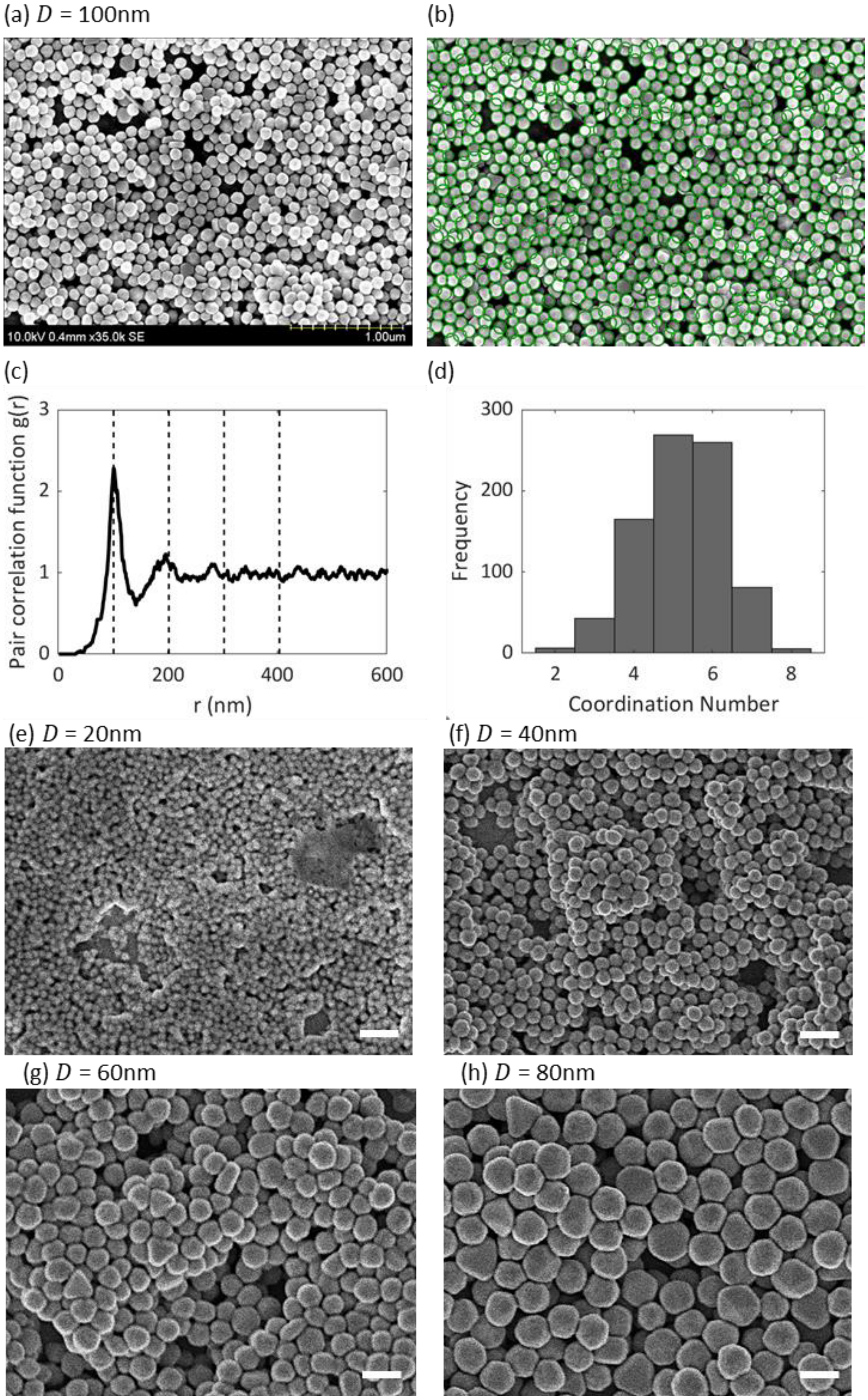

Figure S1. Nanoparticle structural analysis. (a) SEM image of AuNP diameter $\boldsymbol{D}$=100nm 1ML and 2ML regions. (b) Centred circles on each nanoparticle extracted using Hough transform. (c) Pair correlation function of nanoparticle centres for 2ML 100 nm AuNP sample. (d) Coordination number of nanoparticle clusters within a diameter of 130 nm. SEM images of AuNP NP3ML films of varying nanoparticle sizes: (e) $\boldsymbol{D}$ = 20nm, (f) $\boldsymbol{D}$ = 40nm, (g) $\boldsymbol{D}$ = 60nm, & (h) $\boldsymbol{D}$ = 80nm. Scale bar indicates 100 nm.

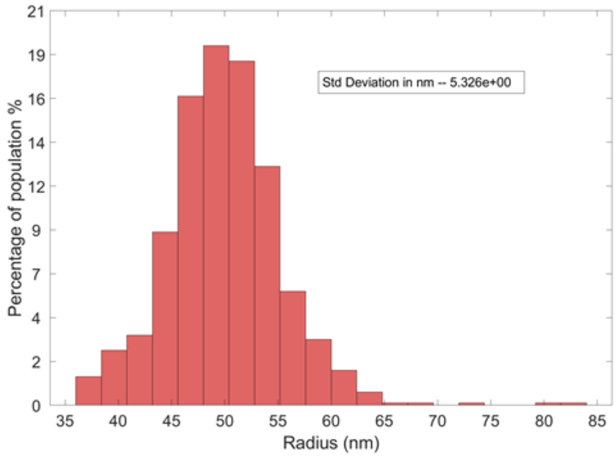

Figure S2. Nanoparticle size distribution analysis from scanning electron microscopy image of 2ML, 100 nm AuNP image in Figure S1a.

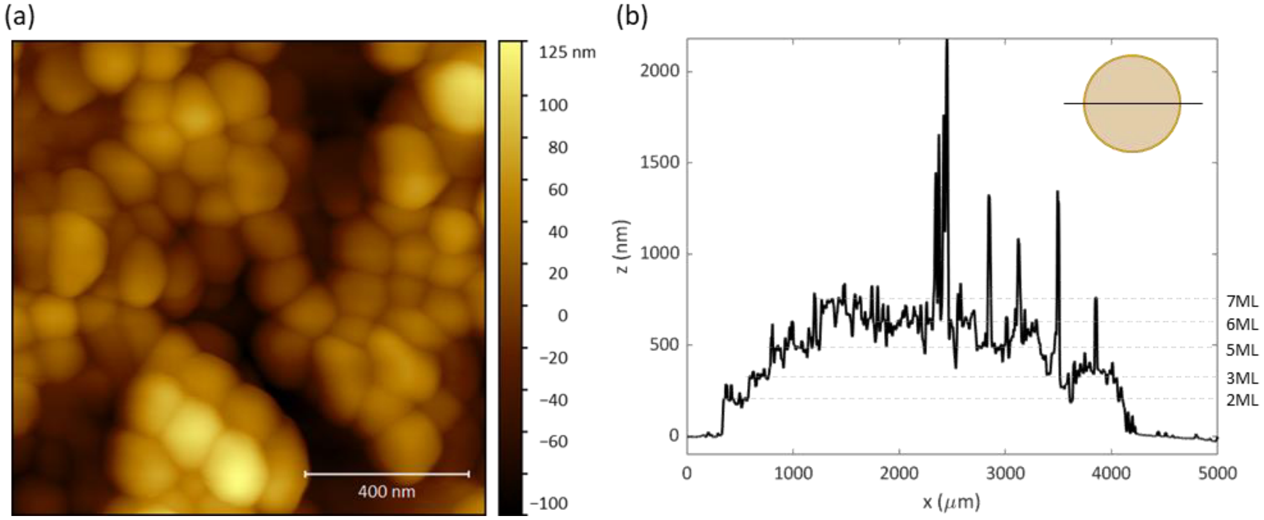

Figure S3. (a) AFM image of 2ML film of 100 nm AuNPs. (b) Dektak profilometer scan of height profile across three consecutive AuNP aggregate depositions on a mirror (NP3ML), with dashed lines indicating number of monolayers in each region.

Section S2: Optical properties of films aggregated with HCl, NaCl, and CB[5]

Films aggregated with 1M aqueous HCl, 1M aqueous NaCl or 1mM CB[5], with the same volumes as the recipe used in the methods section of the main paper, show very different infrared spectra. The NaCl- and HCl-aggregated films show more disordered (lower quality factor) and redshifted ω_{BPP} plasmonic peaks compared to the CB[5] aggregated AuNPs. This confirms the need for a high control of gap size homogeneity in obtaining strong MIR resonances. The homogeneity of CB[5]-scaffolded nanogaps was extensively investigated by transmission electron microscopy[1] and optical dark-field spectroscopy[2] in previous works.

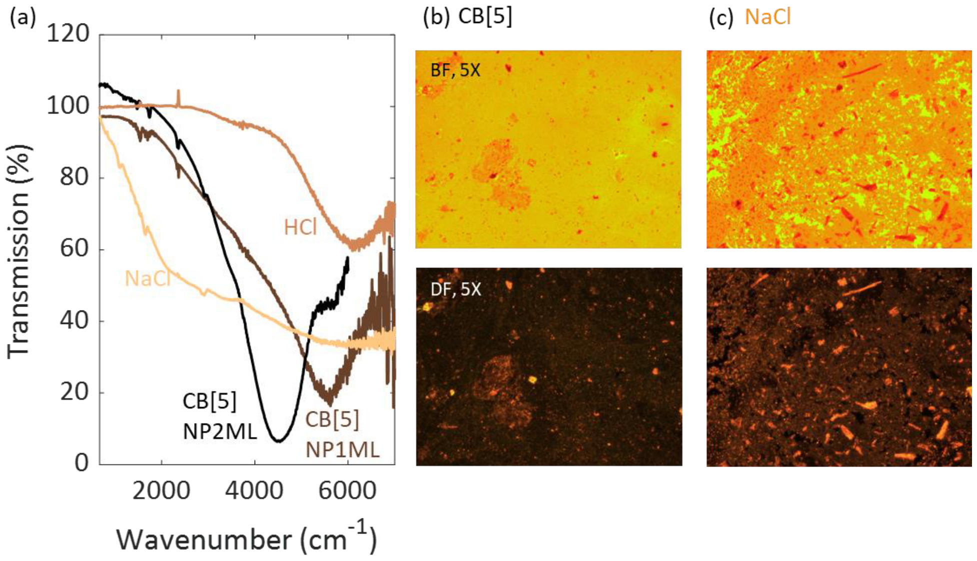

Figure S4. (a) Infrared reflectance spectra of bilayer AuNP on film (NP2ML) samples aggregated with CB[5], NaCl, and HCl. Optical brightfield (BF) and darkfield (DF) images (5x objective lens) of bilayer samples aggregated with (b) CB[5] and (c) NaCl. Strong inhomogeneities visible in the NaCl aggregated sample.

Section S3: Coupled-oscillator effective metamaterial model

The SEIRA spectra and the plasmonic modes of the NPnML films can be understood using a macroscopic dielectric function approach coupled to a single-antenna coupled oscillator mode [3]. This approach adopted by Mueller et. al. [4] was used to successfully fit SEIRA spectra from highly ordered superlattices of AuNPs. In the limit of disordered structures, we attempted to simulate the scattering using the same formalism below, which resulted in an increased plasmon damping rate compared to the highly ordered superlattice.

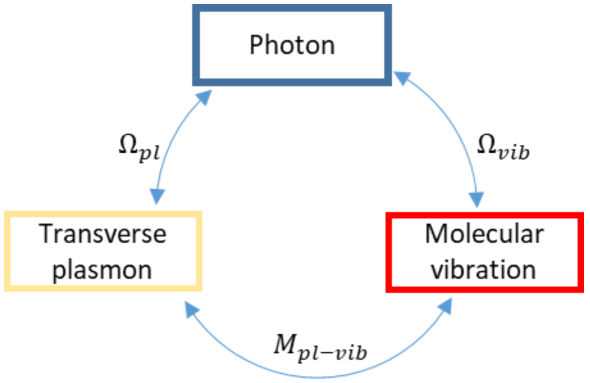

Figure S5. Schematic of coupled oscillators used in effective metamaterial model of SEIRA spectra of NP$\boldsymbol{n}$ML films.

We treat the plasmonic system and molecular vibration as two coupled driven-damped harmonic oscillators represented by the schematic in Figure S5:

$$\frac{d^2}{dt^2}\mu_{\text{pl}}(t) + \omega_{\text{pl}}^2\,\mu_{\text{pl}}(t) + \gamma_{\text{pl}}\frac{\text{d}}{\text{dt}}\mu_{\text{pl}}(t) = 4\Omega_{\text{pl}}^2\left(\epsilon_0\epsilon_{\text{b}}E_0\text{e}^{\text{-i}\omega\text{t}} + M_{\text{pl-vib}}\,\mu_{\text{vib}}(t)\right), \qquad (1)$$

$$\frac{d^2}{dt^2}\mu_{\text{vib}}(t) + \omega_{\text{vib}}^2\,\mu_{\text{vib}}(t) + \gamma_{\text{vib}}\frac{d}{dt}\mu_{\text{vib}}(t) = 4\Omega_{\text{vib}}^2\left(\epsilon_0\epsilon_{\text{b}}E_0\text{e}^{\text{-i}\omega\text{t}} + M_{\text{pl-vib}}\,\mu_{\text{pl}}(t)\right), \qquad (2)$$

where

$\mu_{\text{pl}}, \mu_{\text{vib}}$ = polarization density

$\Omega_{\text{pl}}, \Omega_{\text{vib}}$ = light-matter coupling strength in rad s^{-1}

$M_{\text{pl-vib}}$ = plasmon-molecule coupling factor (dimensionless)

E_0 = amplitude of driving photon field (can be also vacuum field!)

$\omega_{\text{pl}}, \omega_{\text{vib}}$ = frequencies of uncoupled modes

$\gamma_{\text{pl}}, \gamma_{\text{vib}}$ = non-radiative damping

ϵ_{b} = background dielectric constant

These coupled equations can be solved by making the ansatz that the solution is harmonic and takes the form of plane waves, $\mu_{\text{pl}}(t) = \mu_{\text{pl}}^0\,\text{e}^{\text{-i}\omega\text{t}}$ and $\mu_{\text{vib}}(t) = \mu_{\text{vib}}^0\,\text{e}^{\text{-i}\omega\text{t}}$, which leads to the steady-state solutions:

$$\mu_{\text{pl}}^{0}(\omega) = -\frac{4E_0\epsilon_0\epsilon_{\text{b}}\Omega_{\text{pl}}^2\left(1-4\Omega_{\text{vib}}^2\, M_{\text{pl-vib}}/B_{\text{vib}}(\omega)\right)}{B_{\text{pl}}(\omega)-16\,\Omega_{\text{pl}}^2\Omega_{\text{vib}}^2\, M_{\text{pl-vib}}^2\,/B_{\text{vib}}\,(\omega)}, \tag{3}$$

and

$$\mu_{\text{vib},j}^{0}\,(\omega) = -\frac{4E_0\epsilon_{\text{b}}\Omega_{\text{vib}}^2\left(1-4\Omega_{\text{pl}}^2 M_{\text{pl-vib}}/B_{\text{pl}}(\omega)\right)}{B_{\text{vib}}\,(\omega)-16\Omega_{\text{pl}}^2\Omega_{\text{vib}}^2\, M_{\text{pl-vib}}^2/B_{\text{pl}}(\omega)}, \tag{4}$$

where $B_{\text{pl}}(\omega) = \omega^2 - \omega_{\text{pl}}^2 + i\,\gamma_{\text{pl}}\omega$ and $B_{\text{vib}}(\omega) = \omega^2 - \omega_{\text{vib}}^2 + i\,\gamma_{\text{vib}}\omega$.

From the steady-state solutions μ_{pl}^0, and μ_{vib}^0 for the plasmonic and vibrational polarization densities we obtain the linear optical polarizability of the plasmonic crystal $\alpha(\omega) = \left(\mu_{\text{pl}}^0 + \mu_{\text{vib}}^0\right)/E_0$ and the effective dielectric function of the entire NPnML film,

$$\epsilon(\omega) = \epsilon_{\text{b}} + \alpha(\omega)/\epsilon_0 = \epsilon_{\text{b}}\left(1 - \frac{4\Omega_{\text{pl}}^2\left(1-\frac{4\Omega_{\text{vib}}^2 M_{\text{pl-vib}}}{B_{\text{vib}}(\omega)}\right)}{B_{\text{pl}}(\omega)-\frac{16\,\Omega_{\text{pl}}^2\Omega_{\text{vib}}^2 M_{\text{pl-vib}}^2}{B_{\text{vib}}(\omega)}} - \frac{4\Omega_{\text{vib}}^2\left(1-\frac{4\Omega_{\text{pl}}^2 M_{\text{pl-vib}}}{B_{\text{pl}}(\omega)}\right)}{B_{\text{vib}}(\omega)-\frac{16\Omega_{\text{pl}}^2\Omega_{\text{vib}}^2 M_{\text{pl-vib}}^2}{B_{\text{pl}}(\omega)}}\right), \tag{5}$$

For multiple vibrations *j* = 1, …, *N* this gives:

$$\frac{d^2}{dt^2}\mu_{\text{pl}}(t) + \omega_{\text{pl}}^2\,\mu_{\text{pl}}(t) + \gamma_{\text{pl}}\frac{\text{d}}{\text{dt}}\mu_{\text{pl}}(t) = 4\Omega_{\text{pl}}^2\left(\epsilon_0\epsilon_{\text{b}}E_0\text{e}^{\text{-i}\omega\text{t}} + M_{\text{pl-vib}}\,\sum_{j=1}^{N}\mu_{\text{vib},j}\,(t)\right), \tag{6}$$

$$\frac{d^2}{dt^2}\mu_{\text{vib},1}(t) + \omega_{\text{vib},1}^2\,\mu_{\text{vib},1}(t) + \gamma_{\text{vib},1}\frac{d}{dt}\mu_{\text{vib},1}(t) = 4\Omega_{\text{vib},1}^2\left(\epsilon_0\epsilon_{\text{b}}E_0\text{e}^{\text{-i}\omega\text{t}} + M_{\text{pl-vib}}\,\mu_{\text{pl}}(t)\right), \tag{7}$$

…

$$\frac{d^2}{dt^2}\mu_{\text{vib},N}(t) + \omega_{\text{vib},N}^2\,\mu_{\text{vib},N}(t) + \gamma_{\text{vib},N}\frac{d}{dt}\mu_{\text{vib},N}(t) = 4\Omega_{\text{vib},N}^2\left(\epsilon_0\epsilon_{\text{b}}E_0\text{e}^{\text{-i}\omega\text{t}} + M_{\text{pl-vib}}\,\mu_{\text{pl}}(t)\right), \tag{8}$$

This leads to

$$\epsilon(\omega) = \epsilon_{\text{b}}\left(1 - \frac{4\Omega_{\text{pl}}^2\left(1-\sum_{j=1}^{N}\frac{4\Omega_{\text{vib},j}^2\, M_{\text{pl-vib}}}{B_{\text{vib},j}\,(\omega)}\right)}{B_{\text{pl}}(\omega)-\sum_{j=1}^{N}\frac{16\,\Omega_{\text{pl}}^2\Omega_{\text{vib},j}^2\, M_{\text{pl-vib}}^2}{B_{\text{vib},j}\,(\omega)}} - \sum_{j=1}^{N}\frac{4\Omega_{\text{vib},j}^2\left(1-\frac{4\Omega_{\text{pl}}^2 M_{\text{pl-vib}}}{B_{\text{pl}}(\omega)}\right)}{B_{\text{vib},j}\,(\omega)-\frac{16\Omega_{\text{pl}}^2\Omega_{\text{vib},j}^2\, M_{\text{pl-vib}}^2}{B_{\text{pl}}(\omega)}}\right). \tag{9}$$

To calculate the SEIRA spectra we treat the plasmonic NPnML film as a metamaterial with effective refractive index $n(\omega) = \sqrt{\epsilon(\omega)}$ and thickness $h = N_L\sqrt{2/3}(d+a)$ (assuming an fcc stacking), where N_L is the number of stacked nanoparticle layers, d the nanoparticle diameter and a the interparticle gap size.

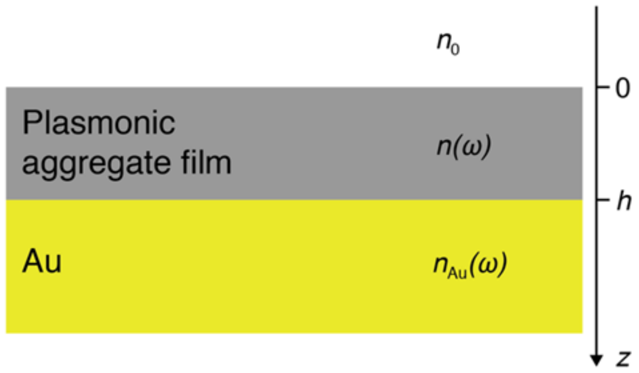

Figure S6. Schematic layer model representing NP$\boldsymbol{n}$ML films on top of a gold mirror.

The transfer matrix for this geometry is

$$\overleftrightarrow{M} = \frac{1}{t_{01}t_{12}} \begin{pmatrix} 1 & r_{01} \\ r_{01} & 1 \end{pmatrix} \begin{pmatrix} e^{i\,k\,h} & 0 \\ 0 & e^{-i\,k\,h} \end{pmatrix} \begin{pmatrix} 1 & r_{12} \\ r_{12} & 1 \end{pmatrix}, \tag{10}$$

with

$$r_{01} = \frac{n_0 - n(\omega)}{n_0 + n(\omega)}, \quad r_{12} = \frac{n(\omega) - n_{Au}(\omega)}{n(\omega) + n_{Au}(\omega)}, \quad t_{01} = \frac{2n_0}{n_0 + n(\omega)}, \quad t_{12} = \frac{2n(\omega)}{n(\omega) + n_{Au}(\omega)}, \tag{11}$$

and $k = n(\omega)\ \omega/c$. The complex reflectivity is

$$r = \frac{M_{21}}{M_{11}} = \frac{r_{01}e^{ikh} + r_{12}e^{-ikh}}{e^{ikh} + r_{01}r_{12}e^{-ikh}} \tag{12}$$

and the reflection is calculated as $R = |r|^2$.

The resulting fit is shown in Figure S7a below for a set of molecular vibrations in the 7ML films. The real and imaginary parts of the effective refractive index are also shown in Figure S7b, highlighting the large real effective refractive index of the films. The fit parameters used for Figure S7a are:
h = 315 nm, $M_{\text{pl-vib}}$ = 0.7, ϵ_∞= 1.5,
ω_{pl} = 1.2 eV, Ω_{pl} = 1.7 eV, γ_{pl} = 0.95 eV,
ω_{vib} = 1740 cm^{-1}, Ω_{vib} = 38 cm^{-1}, γ_{vib} = 30 cm^{-1}
ω_{vib} = 1600 cm^{-1}, Ω_{vib} = 35 cm^{-1}, γ_{vib} = 80 cm^{-1}
ω_{vib} = 1480 cm^{-1}, Ω_{vib} = 30 cm^{-1}, γ_{vib} = 30 cm^{-1}

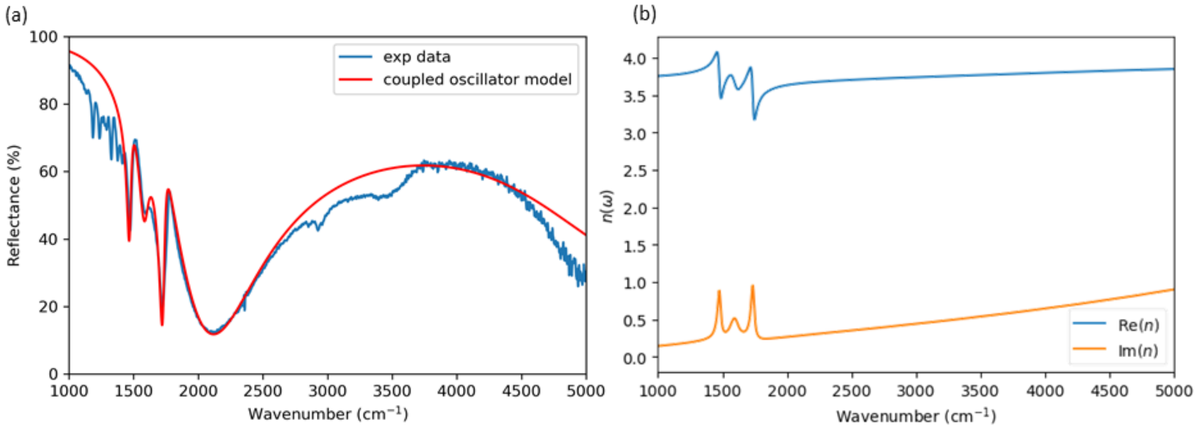

Figure S7. (a) Effective metamaterial model fit to SEIRA spectra from NP$\mathbf{7}$ML films. (b) Complex refractive index extracted from effective metamaterial model fit.

The key difference in the fit between the amorphous NPnML films and the equivalent highly ordered superlattice is the magnitude of the plasmonic oscillator damping γ_{pl} = 0.95 eV, which is twice the plasmon damping in the Drude model for bulk gold ($\gamma_{\mathrm{pl,Drude}}$ = 0.45) [5, 6]. We attribute this to the disorder in the arrangement of AuNPs forming the NPnML films. We can see this in Figure S8a where, as the disorder is increased from γ_{pl} from 0.05 eV to 1.2 eV, the higher order plasmonic modes broaden. However, the lowest order plasmonic mode around 2000cm^{-1} does not broaden and instead becomes much more prominent as the damping is increased. This can be attributed to the increased damping only slightly decreasing the real part of the effective refractive index (Figure S8c) while greatly increasing the imaginary part in the infrared (< 5000 cm-1), thus increasing the dip strength of the fundamental plasmonic mode. At low energies, the mode linewidths are set by the radiative losses (related to the real refractive index), while its strength is set by the absorptive losses (related to the imaginary refractive index). A further contribution is the broadening and overlapping of the higher order modes lending spectral weight to the lowest order plasmonic mode.

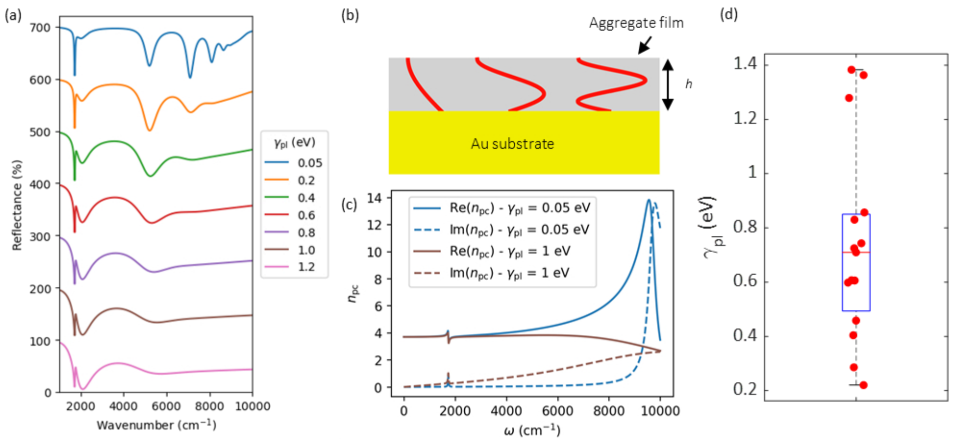

Figure S8. (a) Model reflectance spectrum of NP$\mathbf{7}$ML films vs plasmonic damping parameter $\boldsymbol{\gamma_{pl}}$ from 0.05 eV to 1.2 eV. (b) Schematic of modes within the NP$\boldsymbol{n}$ML films on a gold mirror. (c) Complex refractive index for NP7ML

films with $\boldsymbol{\gamma_{pl}} = 0.05$ eV and 1 eV. All other parameters as in Figure S7. (d) $\boldsymbol{\gamma_{pl}}$ values fitted for a range of NP5ML to NP7ML films.

The fit parameters for the remaining multilayers in Fig 1e can also be found below, with each entry corresponding to a certain layer number:

N = [1, 2, 3, 4, 5, 6, 7, 8, 9] = layer number

$L = \sqrt{\frac{2}{3}}ND$ = Thickness of the NPnML film with nanoparticles of diameter, D = 100 nm.

Ω_{pl} = [1, 0.8, 0.84, 0.84, 0.85, 0.83, 0.9, 1.1, 1.2] eV

ω_{pl} = [0.89, 0.9 ,0.98 ,1 ,0.99, 1, 1, 0.95, 0.95]

γ_{pl} = [0.3, 0.25, 0.65, 0.8, 1.1, 1.3, 1, 1.4, 1.8]

ϵ_{∞}= [1.3^2, 1.3^2, 1.3^2, 1.3^2, 1.3^2, 1.3^2, 1.3^2, 1.3^2, 1.3^2]

The plasmonic modes can also be identified as bulk-plasmon polariton modes from the interference of the collective plasmonic dipoles in the lattice with the propagating field of light (details in the main text). From there, we can obtain the predicted new plasma frequency of the bulk plasma polariton as 0.74 eV, which can be independently predicted from a generalized coupled circuit model.

The size of the nanogap influences the value of the collective plasmon energy $\widetilde{\omega}_{\mathrm{p}}$, see Eqn. (1) of the main text. The modes that we observe in Fig. 1d,e are due to the plasmon-polaritons (mixed light-plasmon excitations) that form standing waves in the gold nanoparticle multilayers. Their dispersion depends more on the volume density of nanoparticles (Ref. [18]) than on the interparticle gaps. The polariton dispersion originates from an anti-crossing of the collective plasmon mode $\widetilde{\omega}_{\mathrm{p}}$ and photons ω_l within the material (as explained in the main text and Refs. [18] and [26]). The resulting collective plasmon-polariton dispersion ω_{BPP} then can only be accessed at integer multiples of the standing waves of light within the material.

A simple estimate of the 1ML plasmon frequency in the small gap limit, is given by twice the dimer red-shift (as given by nearest neighbour coupling on an infinite chain), and can be determined from a generalized circuit model[7]. This indicates that the dominant interaction accounting for the mode redshifting is the capacitance of the AuNP nanogaps. A simple estimate given by the circuit model is:

$$\left(\frac{\widetilde{\omega}_{\mathrm{p}}}{\omega_{\mathrm{Au}}}\right)^{-2} \simeq \varepsilon_{\infty} + 2n_{\mathrm{eff}}^2 + 8\, n_{\mathrm{g}}^{2.6} n_{\mathrm{eff}}^2\, log\left(1 + 0.08\, \frac{D}{d}\right) \qquad (13)$$

with refractive index inside the nanogaps n_{g}~1.45, n_{eff}~2.3, ε_{∞}=10.4, and the bulk Au plasmon ω_{Au}=8.4 eV in the Drude model. From this expression, as the nanogap spacing d decreases, the collective plasmon energy $\widetilde{\omega}_{\mathrm{p}}$ decreases, and thus the anticrossing will occur at a lower energy. However, if we double the gap size from d = 0.9 nm to d = 2 nm, $\widetilde{\omega}_{\mathrm{p}}$ only changes by 10% from ~0.73 eV to ~0.8 eV. Agreement of the resulting estimate $\widetilde{\omega}_{\mathrm{p}}$=0.73 eV for 100nm NPs to the fit from the data

shows that along with equation (2) in the main text, this provides a simple way to design the BPP mode for particular vibrational resonances.

Section S4: FDTD simulations of ordered AuNP superlattices and near fields

The scattering spectrum of the AuNP NPnML layers on a mirror can also be approximated in the highly ordered limit by simulating a face-centered cubic multilayer of 100 nm AuNPs on a mirror using periodic boundary conditions in a finite-difference time-domain solver (Lumerical). We constructed the unit cell of a hexagonal lattice of spherical gold nanoparticles in the *xy* plane and used periodic boundary conditions for the simulation cell. A second layer was placed on top with the *ab* stacking sequence of a hexagonally close-packed structure. The gold nanoparticles had diameters of 100 nm and were spaced apart by 0.9 nm interparticle gaps. The structure was then placed 1.3 nm above a gold mirror. We used 0.3 nm cubic mesh cells to discretize space around the nanoparticles. The optical response of gold was modelled with a fit of experimental data for the dielectric function of gold by Olmon et al.[8]. The nanoparticles were surrounded by a medium with refractive index 1.45 to mimic the dielectric environment from the ligand molecules. The structure was illuminated by a 3 fs light pulse that was emitted from a plane-wave source along z. The reflected light was recorded with a power monitor positioned behind the light source and the reflection spectrum obtained by a Fourier transformation. Electric field monitors were positioned in the gaps between neighbouring nanoparticles to obtain the electric field enhancement in the hotspots. Additionally, we used a 2D electric field monitor between the nanoparticle layers and the gold mirror to obtain the spatial profile of the field enhancement.

The observed reflectance spectrum in Figure S9a is very similar to that predicted by the effective metamaterial model, and corresponds to the geometry shown in Figure S9b. The optical near field amplitude is high at the location of each of the far-field plasmonic resonances, and shows a Fano-like characteristic which differs between the top and bottom layers (Fig.S9c). The fundamental plasmonic mode has an anti-node near the mirror surface, while the midsection of the bottom layer has a lower near-field amplitude than the top layer. There is also a slight spectral shift between the near-field resonance and far-field resonance, with the far-field resonance in the reflectance spectrum at lower energies than the near-field resonance.

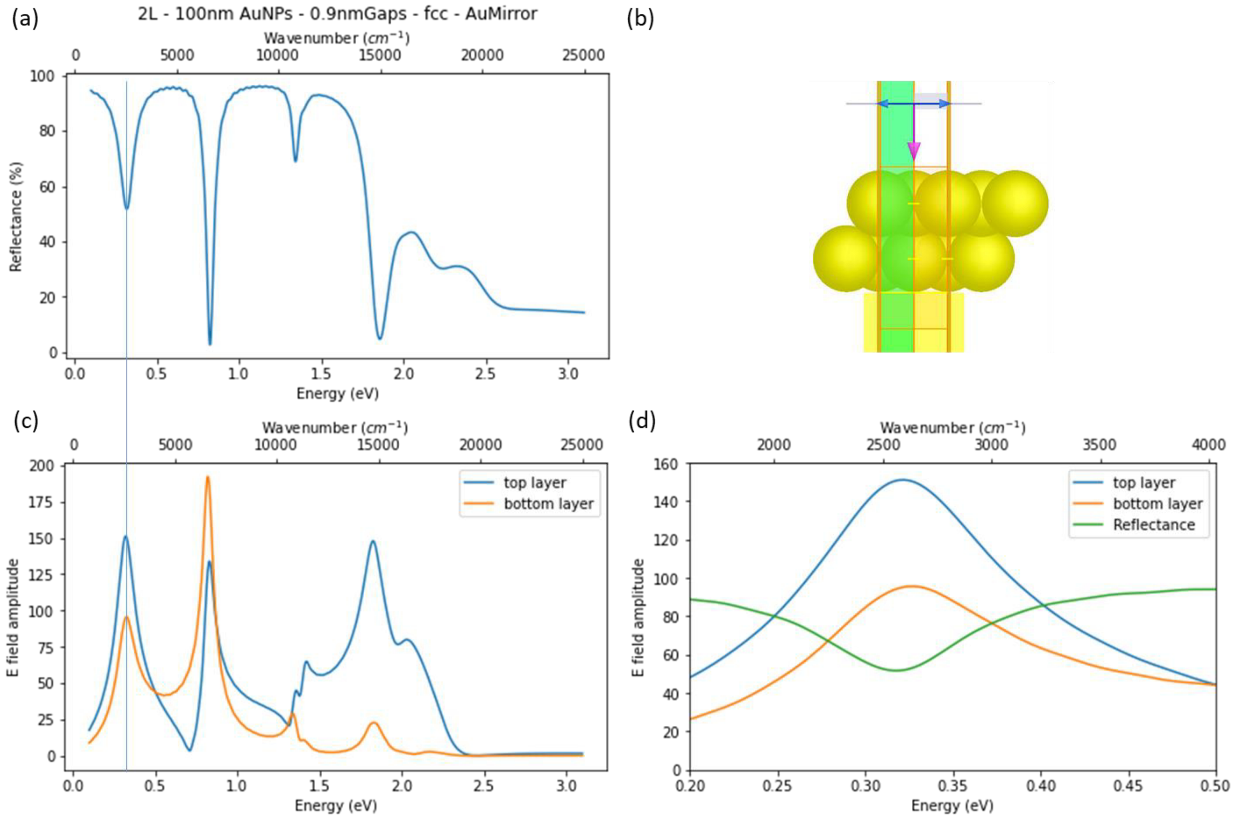

Figure S9. FDTD simulations of face-centred cubic (fcc) AuNP multilayers. (a) Bilayer 100 nm AuNP-on-mirror reflectance spectrum. (b) Schematic of simulated fcc bilayer and FDTD simulation cell. (c) Near field maximum amplitude (relative to normally-incident plane wave) at the midsection of the top layer and the midsection of the bottom layer. (d) Expanded plot of the near field amplitude in (c) showing spectral shift in lowest plasmonic mode in the near-field spectrum vs the far-field reflectance spectrum.

In Fig. S10 we plot a near field map to show the field localization at the interface between the bilayer and the mirror. This Figure demonstrates the low magnitude of field enhancement in the infrared plasmonic resonance (maximum $E \sim 3$) and in the visible (maximum $E \sim 7$). It thus indicates that in the perfectly periodic ordered AuNP superlattice, the field enhancement and hence SEIRA and SERS of molecules in the gap between the mirror and the multilayers is low (Figure S11). However, based on experimental measurements in Fig.4 of the main text, the SERS enhancement of a 4′-cyanobiphenyl-4-thiol self-assembled monolayer in the gap between the mirror and NPnML films remains high, which we attribute to disorder-enabled coupling of light, as discussed below.

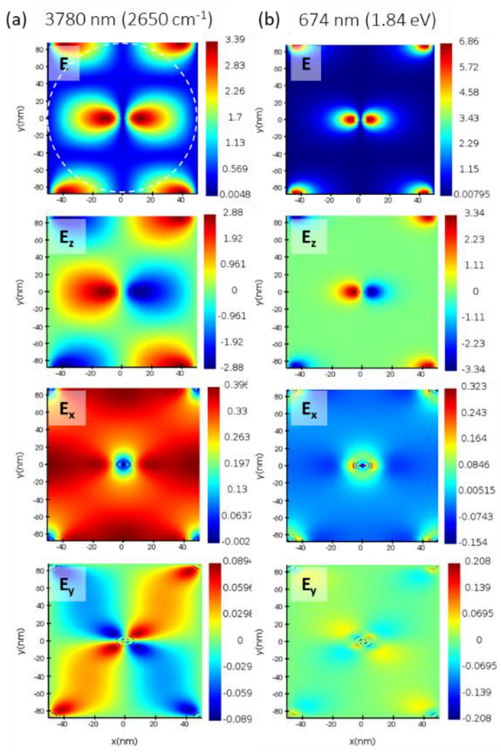

Figure S10. Vector components of the near-field amplitudes relative to normal incident plane wave excitation (magnitude of E and its $\boldsymbol{x}$-, $\boldsymbol{y}$-, and $\boldsymbol{z}$-components) for the fundamental plasmonic mode at $\boldsymbol{\lambda}$=3780 nm, and higher-order modes at $\boldsymbol{\lambda}$=674 nm, sliced through the centre of the bottom interface between the AuNP bilayer and the gold mirror. Dashed circle in (a) is NP equator.

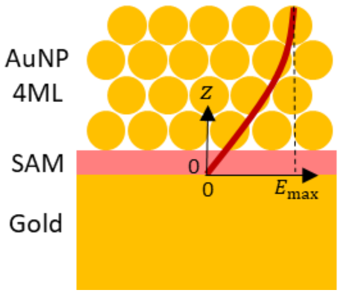

Figure S11. NP$\mathbf{4}$ML on mirror schematic showing the electric field ($\boldsymbol{x}$-component, $\boldsymbol{E_x}$) localization in the vertical ($\boldsymbol{z}$) direction as a quarter-wave, with an antinode at the top surface and a node at the bottom surface on the mirror.

We performed additional periodic FDTD simulations of a supercell with a defect (Figure S12, for 60 nm AuNPs to reduce the computational expense). The near-field for laser excitation wavelength (λ = 633 nm) at the location of NPs around the vacancy defect is $E_z/E_{0,z}$~ 11 giving ten-fold larger intensities than in the perfectly ordered superlattice ($\left|E_z/E_{0,z}\right|^2$~120 in the disordered supercell and ~14 in the ordered superlattice at 633 nm). A similar field enhancement is observed for the infrared resonance (λ = 2370 nm). This shows that a defect will increase the coupling of light into the plane between the NPnML layer and the mirror, but does not disturb the resonant mode.

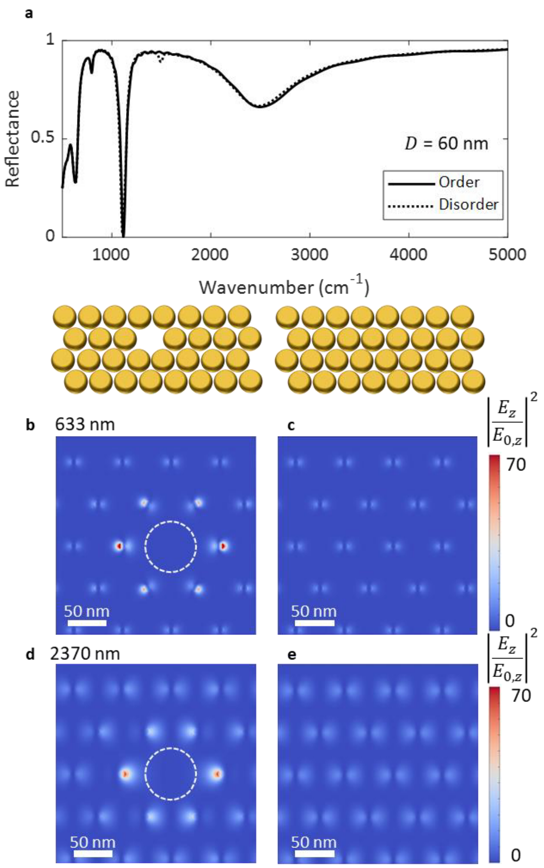

Figure S12. Supercell FDTD simulations. (a) Reflectance spectrum of bilayer of AuNPs on a mirror with perfect order, and of 4x8 AuNP supercell with AB stacking on a mirror and single nanoparticle vacancy at the bottom layer closest to mirror. Vertical near-field intensity ($\left|\boldsymbol{E_z/E_{0,z}}\right|^2$) enhancements at $\boldsymbol{\lambda}$ = 633 nm for (b) 4x8 disordered supercell, (c) perfectly ordered layer; and at $\boldsymbol{\lambda}$ = 2370 nm for (d) 4x8 disordered supercell, and (e) perfectly ordered layer.

In order to optimize the SEIRA enhancement and increase the limit of detection, the near-field intensity ($\left|E_z/E_{0,z}\right|^2$) was investigated as a function of nanoparticle diameter, while adjusting the layer number to keep the resonant ω_{BPP} positions in the same spectral range (Figure S13b). NPnMLs made of AuNPs with larger diameters (D) provide the largest near field enhancement in the hotspots within the layers (Figure S13c). However, the NPnMLs with the largest nanoparticle diameters have the smaller density of hotspots. When correcting the enhancement by the density of hotspots within each layer ($\rho_{hotspot}$) and the number of layers (n), NPnMLs of the smallest nanoparticles (D=25 nm) are found to provide the largest enhancement [Figure S13d]. For D=25 nm AuNPs, these however require 8ML to achieve similar resonances to a bilayer of D=100 nm AuNPs.

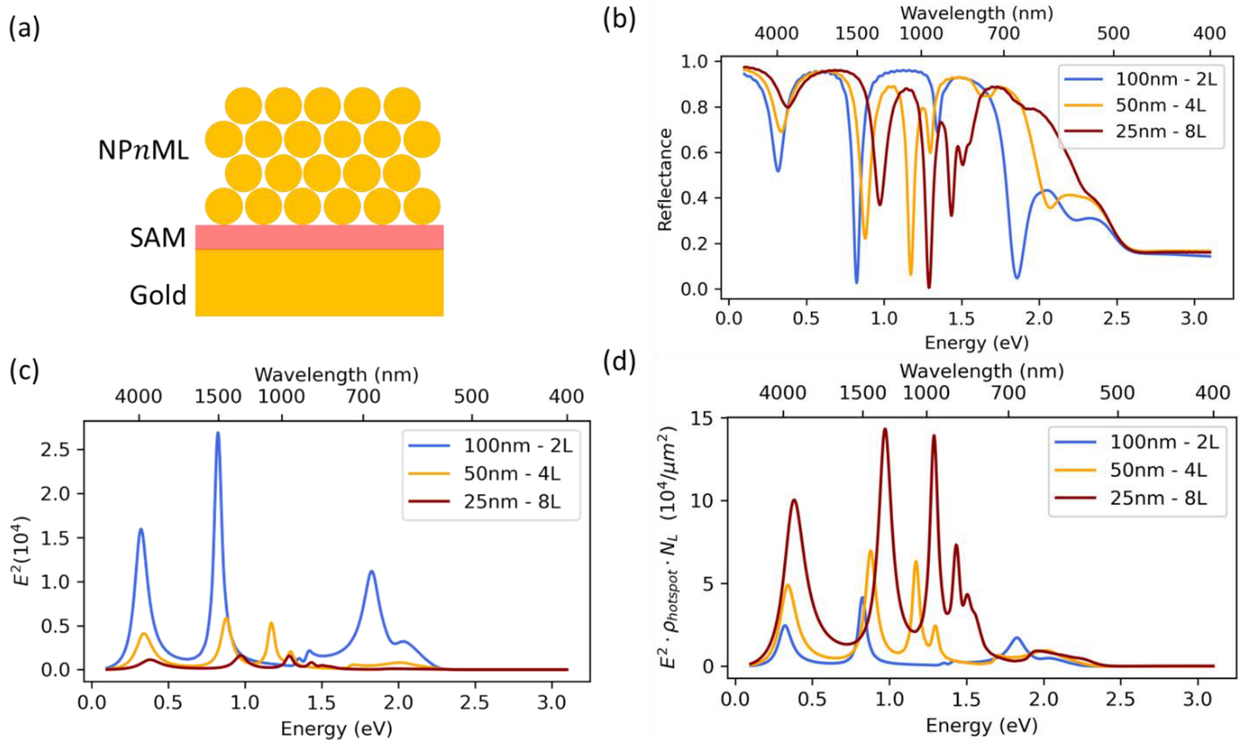

Figure S13. SEIRA enhancement with varying NP size. (a) Schematic of NPnML film. (b) Reflectance spectrum of NPnML films *vs* nanoparticle diameter ($\boldsymbol{D}$ = 25, 50, 100 nm) with number of layers ($\boldsymbol{n}$ = 2, 4, 8) varied to maintain approximate resonance positions. (c) Average near-field enhancement spectrum per hotspot and (d) average near field enhancement spectrum, scaled by number density of hot-spots.

Section S5: Assignment of CB[5] infrared and Raman modes

These are assigned from density functional theory calculations and Refs. [9] and [10]

CB[5]	Infrared-active assignment
1772	C=O stretch
1505	C-H and C=O rocking
1434	Antisymmetric C-N stretch and antisymmetric C-H bending
1394	Symmetric C-N stretch and symmetric C-H bending
1346	Antisymmetric periphery C-H bending
1309	Symmetric C-N stretch and antisymmetric C-H bending
1258	Antisymmetric C-H bending
1210	Antisymmetric C-H bending
973	Symmetric C-H rocking

CB[5] only	Raman-active assignment
1760	C=O stretch
1638	Hydrogen bonding related interactions possibly [THC REF]
1423	Asymmetric C-N stretch
1379	Symmetric C-N stretch
887	complex ring breathing like mode
829	8-membered ring deforms out-of-phase with the 5-membered glycouril ring

Section S6: Xray photoelectron spectroscopy (XPS) of the monolayer CB[5] aggregated AuNP films

Xray photoelectron spectroscopy was used to determine the surface coverage of CB[5] on the monolayer aggregate films from a survey spectrum (Figure S14a). As CB[5] has molecular formula $C_{30}H_{30}N_{20}O_{10}$, we can use the Au:N atomic fractions to obtain an estimate of the surface coverage. The nitrogen content can be unambiguously associated with the CB[5] molecule (Figure S14b), unlike the oxygen or carbon.

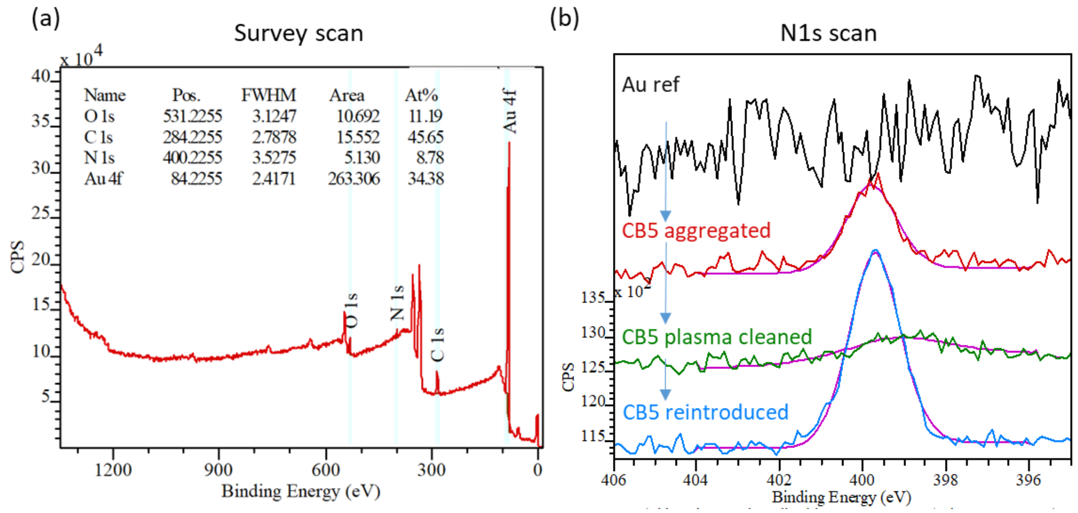

Figure S14. Xray photoelectron spectrum of the monolayer AuNP aggregated with CB[5] then plasma cleaned to remove organic ligands and then CB[5] reintroduced by immersion in 1mM CB[5] solution overnight. (a) Survey spectrum of CB[5] aggregated 1ML AuNPs. (b) N1s spectrum of reference bare gold film, gold film with CB[5] aggregated 1ML deposited (CB[5] aggregated), 1ML post-plasma cleaning to remove CB[5] and other organic ligands (CB[5] plasma cleaned), and 1ML post-plasma cleaning and reintroduction of CB[5] by immersion in 1mM CB[5] solution overnight (CB[5] reintroduced). This shows plasma cleaning removes the CB[5] and thus all N comes from CB[5] on the surface.

From the universal XPS probe depth curve [11], the XPS probes 2.2 nm deep into an Au/C surface (for 0.24 nm interlayer spacing of Au (111) facet[12] this is ~5 gold surface layers probed and 1 nm CB[5]). As the inter-gold atom spacing on Au (111) facet is d_{Au}= (0.288 nm)×cos(30°)=0.249 nm[12] and the lateral size of CB[5] is 1 nm, the highest surface coverage is 1 CB[5] molecule to 3x3 gold surface atoms. The surface number density of gold atoms (ρ_{Au}) is then:

$$\rho_{\mathrm{Au}} = \frac{1}{d_{\mathrm{Au}}^2} = 0.14\ \mathrm{Au\ atoms\ Å^{-2}}$$

Our XPS atomic % ratios then are $\delta_{\mathrm{CB5}} = \frac{A_{\mathrm{N}20}}{A_{\mathrm{Au\ monolayer}}} = \frac{8.78\%/20}{34.4\%/5} = 0.064$, giving a surface coverage of CB[5] of:

$$\rho_{\mathrm{CB5}} = \delta_{\mathrm{CB5}} \cdot \rho_{\mathrm{Au}} = 0.0089\ \mathrm{Å^{-2}} = \ 8.9 \times 10^{17}\ m^{-2}$$

This gives a molecular lattice constant (assuming similar f.c.c. packing) of:

$$d_{\text{CB5}} = \sqrt{\frac{1}{\rho_{\text{CB5}}\cos(30°)}} = 1.14\ nm$$

Section S7: SERS and SEIRA enhancement factor estimation

SEIRA enhancement factor calculation for cucurbit-5-uril:

From the surface coverage of CB[5] determined earlier and the FTIR transmission spectrum of aqueous CB[5] solution (5 mM) measured in a liquid transmission cell with BaF_2 windows and a 6 µm path length (Figure S15), we can estimate the SEIRA enhancement factor. From a 5mM solution in a 6 µm path length cell and 0.5 cm radius windows, we obtain $5 \times \frac{10^{-3} mol}{L} \times 6.02 \times 10^{23} \times \pi \times (0.5\ \text{cm})^2 \times 0.0006\ \text{cm} \times 10^{-3}\frac{L}{\text{cm}^3} = 1.4 \times 10^{15}$ molecules of CB[5] in the liquid cell, with an absorbance of 0.2.

$$N_{\text{IR}} = c \cdot N_{\text{A}} \cdot \pi r^2 \cdot L = = 1.4 \times 10^{15} \text{ molecules of CB[5]}$$

where

N_{IR} = number of molecules probed by IR beam in solution within liquid transmission cell
c = concentration of solution within liquid transmission cell = 5 mM
N_{A} = Avogadro's constant = $6.02 \times 10^{23}\ mol^{-1}$
r = radius of liquid transmission cell windows = 0.5 cm
L = path length of liquid transmission cell = 6 µm

The SEIRA spectrum was measured in an FTIR microscope with the 7ML-on-mirror sample and with a 20 µm square spot size. We estimated a surface coverage of CB[5] of $2.3 \times 10^{17}\ \text{m}^{-2}$, which gives:

$$N_{\text{SEIRA}} = \ 8.9 \times 10^{17}\ \text{m}^{-2} \times (20 \times 10^{-6}\ \text{m})^2 \times 7\ \text{layers} = \ 2.49 \times 10^9 \text{ molecules of CB[5]}$$

in the SEIRA experiment, with a normalized absorbance of 0.43.

This gives a lower limit estimate of the SEIRA enhancement factor, as we assume the entire surface coverage of CB5 contributes to the SEIRA signal, while in actuality only a small fraction of that contained within the gaps between AuNPs will contribute (<5% gap area vs NP surface area).

$$SEIRA\ EF = \frac{A_{\text{SEIRA}}/N_{\text{SEIRA}}}{A_{\text{IR}}/N_{\text{IR}}} = \frac{\frac{0.43 \pm 0.02}{2.49 \times 10^9}}{\frac{0.2}{1.4 \times 10^{15}}} = (1.2 \pm 0.1) \times 10^6$$

where A_{SEIRA} and A_{IR} = absorbances of 1732 cm^{-1} peak for CB[5] in SEIRA NP7ML samples and solution IR measurements respectively.

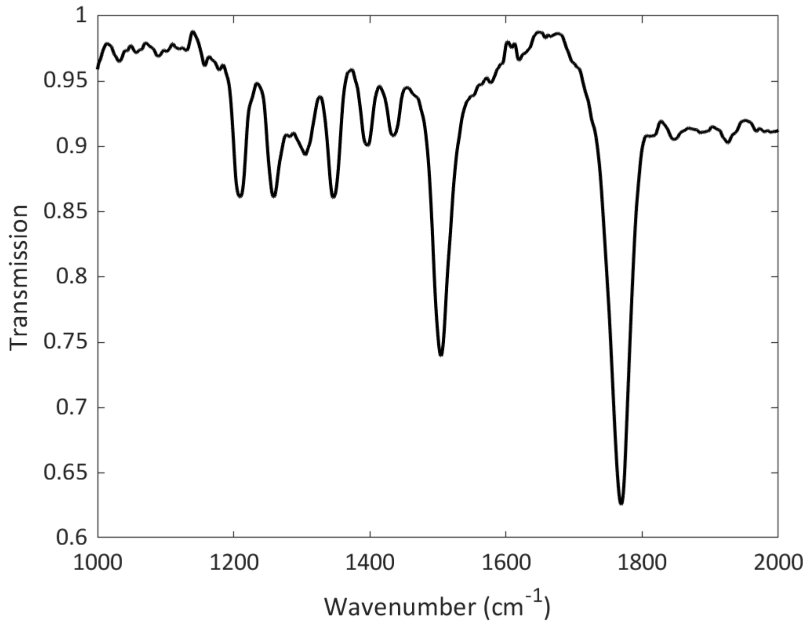

Figure S15. FTIR transmission spectrum of CB[5] aqueous solution (5mM concentration) in a BaF_2 flow cell with 6 µm path length.

SEIRA enhancement factor calculation for 1-decanethiol:

In liquid form, the density of 1-decanethiol is 0.85 g mL^{-1} with a molecular weight of 174.3 g mol^{-1} which yields a number density of $2.9x10^{27}$ m^{-3}. For an IR penetration depth of approximately 10 µm, this yields a surface coverage of $2.9x10^{22}$ m^{-2}. The surface coverage of 1-decanethiol was also estimated from the literature[13], where two phases dominate on Au (111) surfaces, the β phase (0.49 nm x 3.3 nm, centered (23×√3) striped phase) and the δ phase (0.49 nm x 2.2 nm, hexagonal (5 √3x√3) R30°). The β phase and δ phase have similar upper limit surface densities of $6x10^{17}$ and $9x10^{17}$ m^{-2} respectively. To obtain the lower limit of enhancement, we use $9x10^{17}$ m^{-2} as the surface coverage.

$$SEIRA\ EF = \frac{A_{\mathrm{SEIRA}}/\rho_{\mathrm{SEIRA}}}{A_{\mathrm{IR}}/\rho_{\mathrm{IR}}} = \frac{\frac{0.021 \pm 0.02}{9 \times 10^{17}}}{\frac{0.08}{2.9 \times 10^{22}}} = 8.3 \pm 0.1 \times 10^{3}$$

where A_{SEIRA} and A_{IR} = absorbances of ~1460 cm^{-1} peak for 1-decanethiol in SEIRA NP7ML samples and powder IR measurements respectively. ρ_{SEIRA} and ρ_{IR} = surface coverage of 1-decanethiol on the SEIRA substrate and in powder form respectively.

SERS enhancement factor calculation:

BPT-CN (4′-cyanobiphenyl-4-thiol):

For a laser spot size of 1 μm diameter, the number of gaps is <70 (obtained from the spot area divided by the number of 100 nm nanoparticles assuming full coverage and fcc packing). The surface density of the BPT-CN (4′-cyanobiphenyl-4-thiol) self-assembled monolayer is 2.97 nm^{-2} [14], giving an upper bound of 102000 molecules, assuming a facet diameter of 25 nm for the 100 nm AuNPs. The Raman spectrum of BPT-CN at 98 mM in ethanol probes $6.02 \cdot 10^{23} \times 98 \cdot 10^{-3} \times (10^{-4})^3 \times 0.001 = 58996000$ molecules.

$N_{\mathrm{Raman}} = c \cdot N_{\mathrm{A}} \cdot D^3$ = 58996000 molecules of BPTCN

where

N_{Raman} = number of molecules probed by Raman microscope in solution

c = concentration of solution for Raman = 98 mM

N_{A} = Avogadro's constant = $6.02 \times 10^{23}\ mol^{-1}$

D = Raman excitation laser spot size = 1 μm

$N_{\mathrm{SERS}} = \rho_{\mathrm{BPT}} \cdot \pi \left(\frac{D_{\mathrm{facet}}}{2}\right)^2 \cdot n_{\mathrm{gaps}}$ = 102000 molecules of BPTCN

where

ρ_{BPT} = surface number density of BPTCN on gold = 2.97 nm^{-2}

D_{facet} = facet diameter of 100 nm AuNP = 25 nm

n_{gaps} = number of gaps in laser focus = 7 on average.

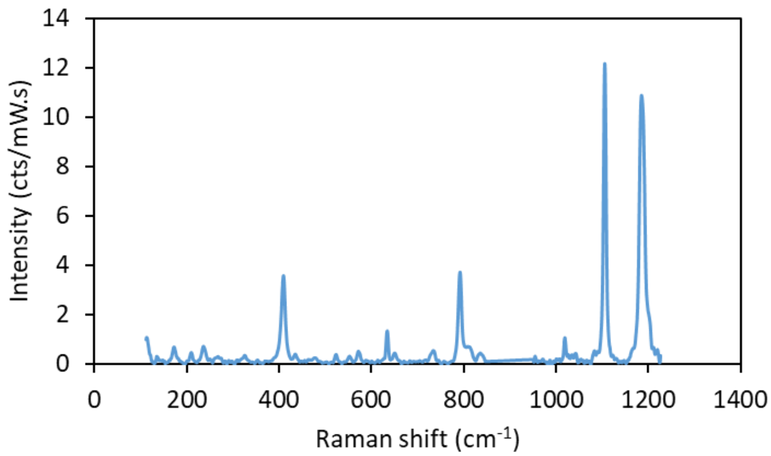

Figure S16. Raman spectrum of BPTCN (4′-cyanobiphenyl-4-thiol) in ethanol (98 mM). Raman excitation laser 785 nm and 127 mW power, 5 s integration time.

Comparing the normalized Raman peak intensity (in counts $mW^{-1}s^{-1}$) with a 785 nm excitation laser for the band at 1188 cm^{-1} gives:

$$SERS\ EF = \frac{I_{\mathrm{SERS}}/N_{\mathrm{SERS}}}{I_{\mathrm{Raman}}/N_{\mathrm{Raman}}} = \frac{\frac{20000\ \mathrm{cts\ mW^{-1}s^{-1}}}{102000}}{\frac{10.7\ \mathrm{cts\ mW^{-1}s^{-1}}}{58996000}} = 1.1 \times 10^{6}$$

where

I_{SERS} and I_{Raman} = Raman intensity of 1188 cm^{-1} peak for CB[5] in SERS NP1ML samples and solution Raman measurements respectively in cts/mW.sec.

1-decanethiol:

In liquid form, the density of 1-decanethiol is 0.85 g mL^{-1} with a molecular weight of 174.3 g mol^{-1} yields a number density of 2.9×10^{27} m^{-3}, for a laser spot size of 1 µm gives:

$N_{\mathrm{Raman}} = \rho_{\mathrm{DT}} \cdot N_{\mathrm{A}} \cdot D^3 = 2.9 \times 10^{9}$ molecules of 1-decanethiol

where

N_{Raman} = number of molecules probed by Raman microscope in solution

ρ_{DT} = concentration of 1-decanethiol liquid = 4876 mol m^{-3}

N_{A} = Avogadro's constant = 6.02×10^{23} mol^{-1}

D = Raman excitation laser spot size = 1 µm

The surface coverage of 1-decanethiol was also estimated from the literature[13], where two phases dominate on Au (111) surfaces, the β phase (0.49 nm x 3.3 nm, centered (23×√3) striped phase) and the δ phase (0.49 nm x 2.2 nm, hexagonal (5 √3x√3) R30°). The β phase and δ phase have similar upper limit surface densities of 6×10^{17} and 9×10^{17} m^{-2} respectively. To obtain the lower limit of enhancement, we will use 9×10^{17} m^{-2} as the surface coverage.

$N_{\mathrm{SERS}} = \rho_{\mathrm{DT}} \cdot \pi \left(\frac{D_{\mathrm{facet}}}{2}\right)^2 \cdot n_{\mathrm{gaps}} = 4948650$ molecules of CB[5]

where

ρ_{DT} = surface number density of 1-decanethiol on gold = 2.97 nm^{-2}

D_{facet} = facet diameter of 100 nm AuNP = 25 nm

n_{gaps} = number of gaps in laser focus = 7 on average.

$$SERS\ EF = \frac{I_{\mathrm{SERS}}/\rho_{\mathrm{SERS}}}{I_{\mathrm{Raman}}/\rho_{\mathrm{SERS}}} = \frac{\frac{3000\ \mathrm{cts\ mW^{-1}s^{-1}}}{4948650}}{\frac{145\ \mathrm{cts\ mW^{-1}s^{-1}}}{2.9 \times 10^{9}}} = 1.2 \pm 0.1 \times 10^{4}$$

where

I_{SERS} and I_{Raman} = Raman intensity of ~1380 cm^{-1} peak for 1-decanethiol in SERS NP1ML samples and solution Raman measurements respectively in cts $mW^{-1}s^{-1}$.

Comparison of SEIRA and SERS enhancement factors (EF) to literature:

The NPnML substrate shows SEIRA enhancements that are the state-of-the-art for self-assembled structures, and compete with expensive lithographically fabricated structures of restricted area. Furthermore, the Fano dip strength (R_{Fano}=40%) exceeds previous record[15] SEIRA transmission dips (36% in 2021).

Table S1. Self-assembled SEIRA substrates

Geometry	Refs.	Field confinement (V) and Q-factor	SEIRA EF (expt)	SERS EF	Analyte	IR source
**NPnML**	-	V = $10^{-8}\lambda^3$<br>Q =2-3	**$1.2x10^6$**	$1.1x10^6$	cucurbit-5-uril	globar
**Au nanowire aggregates**	16	V = (none)<br>Q $<$ 1	$5x10^4$	none	oleylamine	globar
**Au nanoshell arrays**	17-19	V = (none)<br>Q < 1	10^4	10^9	para-mercaptoaniline	globar
**Au nanonails and nanorod aggregates**	20	V = (none)<br>Q $<$ 1	2180	none	4-aminothiophenol	globar
**Ag nanorods with SiO_2 shell**	21	V = (none)<br>Q = 1-2	1000	none	CTAB ligand	globar
**Au nanoparticle multilayers**	22	V = (none)<br>Q $<$ 1	273	none	octadecanethiol	globar
**AgNP and graphene nanoribbons**	23	V = (none)<br>Q = 2-3	170	10^5	Rhodamine-6-g	globar
**Copper nanoparticles**	24	V = (none)<br>Q < 1	40	none	p-nitrobenzoic acid	globar
**Electrochemically deposited Au islands**	25	V = (none)<br>Q $\sim$10	7	none	cytochrome-c	globar
**Au nanostars**	26	V = (none)<br>Q = none	5	$2.5x10^3$	Crystal violet	globar
**AuNP supercrystals**	4	V = (none)<br>Q = 2-3	4	10^2	polystyrene	globar

Table S2. Lithographically fabricated SEIRA substrates

We take experimentally-measured enhancement factors where possible (avoiding simulated values). EFs shown are upper bounds, considering only the active/gap area.

Geometry	Refs.	Field confinement (V) and Q-factor	SEIRA EF (expt)	SERS EF	Analyte	IR source
**Au nanodisk array**	27	V = $2.8x10^{-2}\lambda^3$<br>Q ~5-6	$2.2x10^6$	none	decanethiol	globar
**Au nanorod dimer**	28	V = $10^{-4}\lambda^3$<br>Q $\sim$ 2-3	$2x10^5$	none	4,4'-bis(N-carbazolyl)-1,1'-	synchrotron

					biphenyl	
**Au log-periodic trapezoidal antenna**	[29]	$V = 2x10^{-2}\lambda^3$<br>Q ~5	$2x10^5$	none	Carboxy-terminated alkanethiols	globar
**Au nanoslit Fano metamaterial**	[30]	$V = 6x10^{-5}\lambda^3$<br>Q ~3	$1.6x10^5$	none	PMMA polymer	globar
**Au nanowires - individual**	[31]	$V = 10^{-2}\lambda^3$<br>Q ~ 2-3	10^5	none	octadecanethiol	synchrotron
**Au nanorod array**	[32]	$V = 10^{-6}\lambda^3$<br>Q ~5-7	10^5	500	methylene blue	globar
**Au nanoslits**	[33]	$V = 10^{-3}\lambda^3$<br>Q ~4-8	$9.3x10^4$	none	octadecanethiol	globar
**Au nanorod - array**	[34]	$V = 10^{-4}\lambda^3$<br>Q ~5-6	$7.2x10^4$	none	octadecanethiol	globar
**Au nanorod - individual**	[35]	$V = 10^{-3}\lambda^3$<br>Q ~ 2-3	$5.5x10^4$	none	C_{60}	globar
**Au nanodisk array**	[36]	$V = 10^{-3}\lambda^3$<br>Q ~1	$4x10^4$	none	4-mercaptobenzoic acid	globar
**Au nanowire - array**	[37]	$V = 10^{-4}\lambda^3$<br>Q ~ 3	$2.5x10^4$	none	4,4′-bis(N-carbazolyl)-1,1′-biphenyl	globar
**Au nanorod – array**	[38]	$V = 10^{-1}\lambda^3$<br>Q ~5-6	$1.4x10^4$	none	4,4′-bis(N-carbazolyl)-1,1′-biphenyl	globar
**Au nanorod array (grating)**	[39]	$V = \lambda^3$<br>Q ~5-6	10^4-10^5	none	proteins	globar
**Au coaxial nanoaperture array**	[40]	$V = 3x10^{-6}\lambda^3$<br>Q ~2-3	10^4-10^5	none	silk protein	globar
**Au nanorod – array**	[41]	$V = 10^{-1}\lambda^3$<br>Q ~2-3	1200	none	C_{60}	globar
**Au nanorod-array**	[42, 43]	$V = \lambda^3$<br>Q ~2-3	1000	none	proteins	globar
**Au/Al cross antennae**	[44, 45]	$V = 3x10^{-4}\lambda^3$<br>Q ~3	750<br>(10^4 thy)	none	silica and octadecanethiol	globar
**Au pad-rod nanoantennae**	[46]	$V = 2x10^{-6}\lambda^3$<br>Q = 8-10	297	none	PMMA	globar
**Au nanowire - array**	[47]	$V = 1.4 \times 10^{-5}\lambda^3$<br>Q ~3-4	270	none	hydrogen silsesquioxane	globar
**Graphene nanoribbon**	[48]	$V = 10^{-2}\lambda^3$<br>Q ~4	100	none	PMMA and PVP	globar
**Au nanogap metasurface**	[49]	$V = 6x10^{-5}\lambda^3$<br>Q~ 5-6	66<br>(10^4 thy)	none	protein antibody	globar
**Au nanoslits**	[50]	$V = 10^{-4}\lambda^3$<br>Q ~4-5	40	none	silica	globar
**Au nanorod – array (grating)**	[51]	$V = 4x10^{-3}\lambda^3$<br>Q ~4-5	20	none	PMMA	globar
**Al/Au bowtie antenna array**	[52]	$V = 10^{-4}\lambda^3$<br>Q ~6	10<br>(241 thy)	none	PMMA	globar
**Au nanopatch array**	[53]	$V = 10^{-3}\lambda^3$<br>Q ~12.3	8.14	none	CO_2 gas	globar
**Au Y-shaped nanoantennae**	[54]	$V = \lambda^3$<br>Q ~6	6-7	none	tris(8-hydroxyquinoline)	globar

					aluminum(III)	
**only theoretical estimates available:**						
**Au fan-shaped nanogap antenna**	[55]	$V = 7x10^{-7}\lambda^3$<br>$Q \sim 6\text{-}7$	(10^7 - theory)	none	4-nitrothiophenol	
**Au nanowire - array**	[56]	$V = 10^{-4}\lambda^3$<br>$Q \sim 5\text{-}7$	(10^3 - theory)	none	lactose permease enzyme	globar
**Al grating array**	[57]	$V = 2x10^{-5}\lambda^3$<br>$Q \sim 7\text{-}8$	(10^3- theory)		proline	globar
**Graphene arrays**	[58]	$V = 4x10^{-6}\lambda^3$<br>$Q \sim 4$	(10^3 theory)	none	proteins	globar
**ITO nanorod - array**	[59]	$V = 10^{-1}\lambda^3$<br>$Q \sim 1\text{-}2$	(10^2 - theory)	none	PMMA polymer	globar

Section S8: Modified coupled-oscillator Fano resonance fitting

From the general model of a medium of modified dielectric index in Section S3, we can further analyze the coupling strength using a modified coupled oscillator model for molecule-plasmon coupling. Below, we make the assumption that there is a single cavity plasmonic mode interacting with a molecular vibration. This analysis is more intuitive and allows Fano parameters to be related to near-field strengths via the local Green's functions, optomechanical coupling, and mode volumes within the plasmonic construct. We show that a recent full theoretical model must be applied to our data, to provide suitable spectral fits, involving the self-interaction of molecules via the plasmonic fields.

To a first approximation, SEIRA spectra can be understood using a simple coupled oscillator model [60] which treats the molecular and the plasmonic resonances as oscillators with a mutual coupling factor. This results, for molecular resonances with linewidths much smaller than plasmonic linewidths, in a Fano lineshape representing the interference of a localized vibrational mode with the plasmonic continuum. Following the derivation in Ref. [60] for the semiclassical Maxwell-Bloch equations, we obtain

$$\frac{d^2}{dt^2}\mu_{\mathrm{pl}}(t) + \omega_{\mathrm{pl}}^2\,\mu_{\mathrm{pl}}(t) + \gamma_{\mathrm{pl}}\frac{\mathrm{d}}{\mathrm{dt}}\mu_{\mathrm{pl}}(t) = F_0 + g\left(\frac{\omega_{\mathrm{pl}}\mathrm{d}_{\mathrm{pl}}}{\mathrm{d}_{\mathrm{m}}}\right)\mu_{\mathrm{m}} \tag{14}$$

$$\frac{d^2}{dt^2}\mu_{\mathrm{m}}(t) + \omega_{\mathrm{m}}^2\,\mu_{\mathrm{m}}(t) + \gamma_{\mathrm{m}}\frac{\mathrm{d}}{\mathrm{dt}}\mu_{\mathrm{m}}(t) = g\left(\frac{\omega_{\mathrm{m}}\mathrm{d}_{\mathrm{m}}}{\mathrm{d}_{\mathrm{pl}}}\right)\mu_{\mathrm{pl}} \tag{15}$$

$\mu_{\mathrm{pl}}, \mu_{\mathrm{m}}$ = polarization density for plasmonic resonance and molecular vibration respectively
d_{pl}, d_{m} = transition dipole moment of plasmonic resonance and molecular vibration respectively
ω_{pl}, ω_{m} = angular frequency of plasmonic resonance and molecular vibration respectively
$\gamma_{\mathrm{pl}}, \gamma_{\mathrm{m}}$ = decay rate of plasmonic resonance and molecular vibration respectively
g = coupling strength of plasmonic resonance to molecular vibration
$F_0 = 4\omega_{\mathrm{pl}}\mathrm{d}_{\mathrm{pl}}^2E_0\exp(-i\omega t)$ = driving force of a plane wave of amplitude E_0 and frequency ω

The equations above assume that the quantum fluctuations of the field can be neglected, the dephasing of the plasmon can be ignored as it is slow compared to energy damping, the dephasing of the molecular vibration is faster than the energy decay, the molecule remains in the lowest vibrational state, and that the system is driven by the plasmon oscillator coupled to a plane wave external field. This results in a solution for the scattering cross-section as (neglecting the molecular dipole as $\mu_{\mathrm{m}} \ll \mu_{\mathrm{pl}}$):

$$\mu_{\mathrm{pl}} = \frac{F_0(\omega_{\mathrm{m}}^2-\omega^2-i\omega\gamma_{\mathrm{m}})}{(\omega_{\mathrm{m}}^2-\omega^2-i\omega\gamma_{\mathrm{m}})\left(\omega_{\mathrm{pl}}^2-\omega^2-i\omega\gamma_{\mathrm{pl}}\right)-\omega_{\mathrm{m}}\omega_{\mathrm{pl}}g^2} \tag{16}$$

$$\sigma_{\mathrm{scat}}(\omega) = \omega^4\left|\mu_{\mathrm{pl}}\right|^2 \tag{17}$$

However, the SEIRA spectrum of NPnML films cannot be simply fit with a Fano profile (Figure S17a), as the depth of the Fano dip is much larger than expected from a simple coupled oscillator model between

a localized molecular vibration and a plasmonic resonance. Due to the symmetry of the coupling parameter and the small molecular dipole strength, the impact of the molecule on the plasmonic resonance is a Fano-lineshape dip in the plasmon scattering, however in the measured spectrum the dip corresponding to the molecule has a much stronger extinction than expected. This can be seen as we increase the coupling strength for a plasmonic resonance (ω_{pl} = 1600 cm^{-1}, γ_{pl}= 700 cm^{-1}) and molecular resonance (ω_{m} = 1765 cm^{-1}, γ_{m}= 30 cm^{-1}) from g = 0 to 0.2γ_{pl} (Figure S17b). This discrepancy is surprising if we only consider the molecule and plasmon as harmonic oscillators within the dipole approximation. However, within a plasmonic cavity with tight field confinement, the self-interaction of the molecule within the cavity becomes important and higher order multi-polar effects come into play, as recently theoretically developed in Ref.[61].

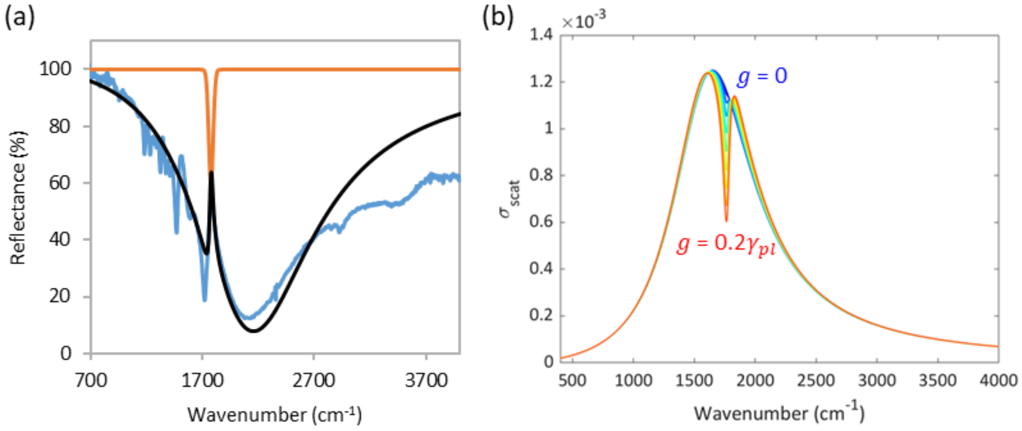

Figure S17. Simple coupled oscillator model of SEIRA. (a) SEIRA spectrum of 7ML AuNP aggregate fitted with simple coupled oscillator model. SEIRA spectrum data (blue), molecular vibrational spectrum (orange), fitted coupled oscillator model (black). (b) Simulation of coupled oscillator model for plasmonic resonance ($\boldsymbol{\omega_{pl}}$ = 1600 cm^{-1}, $\boldsymbol{\gamma_{pl}}$= 700 cm^{-1}) and molecular resonance ($\boldsymbol{\omega_{m}}$ = 1765 cm^{-1}, $\boldsymbol{\gamma_{m}}$= 30 cm^{-1}), vs g ranging from 0 to 0.2$\boldsymbol{\gamma_{pl}}$

If we expand the coupled oscillator model to take into account self-interaction of the molecule, using the treatment within Zhang *et al* [61], we can write the plasmonic (μ_{pl}) and molecular (μ_{m}) dipole moments as:

$$\mu_{\mathrm{pl}} = \alpha_{\mathrm{p}}[E_j(r_{\mathrm{p}},\omega) + \overleftrightarrow{G_0}(r_{\mathrm{p}},r_{\mathrm{m}};\omega)\cdot\mu_{\mathrm{m}}] \quad (18)$$
$$\mu_{\mathrm{m}} = \alpha_{\mathrm{m}}E_{\mathrm{loc}} = \alpha_{\mathrm{m}}[\overleftrightarrow{G_0}(r_{\mathrm{m}},r_{\mathrm{p}};\omega)\cdot\mu_{\mathrm{pl}}] \quad (19)$$

where

α_{pl}, α_{m} = polarizability of the plasmonic and vibrational mode

$E_j(r_{\mathrm{p}},\omega)$ = electric field of incident light as a function of position r_p and frequency ω

$E_{\mathrm{loc}}(r_{\mathrm{p}},r_{\mathrm{m}};\omega)$ = local electric field induced by incident light as a function of position r_{p}, molecular dipole position r_{m} and frequency ω.

$\overleftrightarrow{G_0}(r_{\mathrm{p}},r_{\mathrm{m}};\omega)$ = dyadic Green's function of the plasmonic structure

Zhang *et al* define the corresponding multipolar Green's function of the plasmon and molecular parts as $\overleftrightarrow{G}_{\mathrm{mp}} = \overleftrightarrow{G_0}(r_{\mathrm{m}}, r_{\mathrm{p}}; \omega)$ and $\overleftrightarrow{G}_{\mathrm{pm}} = \overleftrightarrow{G_0}(r_{\mathrm{p}}, r_{\mathrm{m}}; \omega)$, to rewrite the equations above including a molecular dipole self interaction term:

$$\mu_{\mathrm{pl}} = \alpha_{\mathrm{p}}[E_j + \overleftrightarrow{G}_{\mathrm{pm}} \cdot \mu_{\mathrm{m}}] \tag{20}$$
$$\mu_{\mathrm{m}} = \alpha_{\mathrm{m}}[\overleftrightarrow{G}_{\mathrm{mp}}\alpha_{\mathrm{p}}E_j + \overleftrightarrow{G}_{\mathrm{mp}}\alpha_{\mathrm{p}}\overleftrightarrow{G}_{\mathrm{pm}} \cdot \mu_{\mathrm{m}}] \tag{21}$$

They define a field enhancement term $\overleftrightarrow{M} = \overleftrightarrow{G}_{\mathrm{mp}}\alpha_{\mathrm{p}}$ and molecular self-interaction $\overleftrightarrow{G}_{\mathrm{mm}} = \overleftrightarrow{G}_{\mathrm{mp}}\alpha_{\mathrm{p}}\overleftrightarrow{\mathrm{G}}_{\mathrm{pm}}$ to rewrite the generalized coupling equations and obtain the generalized solution for the plasmonic dipole as:

$$\mu_{\mathrm{pl}} = \alpha_{\mathrm{p}}\left[\overleftrightarrow{I} + \frac{\overleftrightarrow{G}_{\mathrm{pm}} \cdot \overleftrightarrow{M}}{\alpha_{\mathrm{m}}^{-1} - \overleftrightarrow{G}_{\mathrm{mm}}}\right]E_j \tag{22}$$

where $\overleftrightarrow{I}$ is the unit dyadic tensor. A residual self-interaction term can be defined, which would be zero within the dipolar approximation as $\overleftrightarrow{G}_{\mathrm{res}} = \overleftrightarrow{G}_{\mathrm{mm}} - \overleftrightarrow{G}_{\mathrm{pm}} \cdot \overleftrightarrow{M}$ to account for all multipolar interactions. Assuming an isotropic molecular and plasmonic polarizability $\alpha_{\mathrm{m}} = \alpha_{\mathrm{m}}^0\omega_{\mathrm{m}}^2/\,(\omega_{\mathrm{m}}^2 - \omega^2 - i\omega\gamma_{\mathrm{m}})$, they rewrite the plasmonic dipole as:

$$\begin{aligned} \mu_{pl} &= \alpha_p\left[\overleftrightarrow{I} + \frac{\alpha_{\mathrm{m}}^0\omega_{\mathrm{m}}^2\overleftrightarrow{G}_{\mathrm{pm}} \cdot \overleftrightarrow{M}}{(\omega_{\mathrm{m}}^2 - \omega^2 - i\omega\gamma_{\mathrm{m}}^0) - \alpha_{\mathrm{m}}^0\omega_{\mathrm{m}}^2\overleftrightarrow{G}_{\mathrm{mm}}}\right]E_j \\ &= \alpha_p\left[\frac{(\omega_{\mathrm{m}}^2 - \omega^2 - i\omega\gamma_{\mathrm{m}}^0) - \alpha_{\mathrm{m}}^0\omega_{\mathrm{m}}^2\overleftrightarrow{G}_{\mathrm{res}}}{(\omega_{\mathrm{m}}^2 - \omega^2 - i\omega\gamma_{\mathrm{m}}^0) - \alpha_{\mathrm{m}}^0\omega_{\mathrm{m}}^2\overleftrightarrow{G}_{\mathrm{mm}}}\right]E_j \end{aligned} \tag{23}$$

Zhang *et al* then defined various terms that represent shifts from the normal dipole approximation:

$$\delta\omega_{\mathrm{L}} = -\frac{\omega_{\mathrm{m}}}{2}\alpha_{\mathrm{m}}^0\Re e[\hat{n}_{\mathrm{m}} \cdot \overleftrightarrow{G}_{\mathrm{mm}} \cdot \hat{n}_{\mathrm{m}}] \tag{24}$$

$$\delta\omega_{\mathrm{L}}' = -\frac{\omega_{\mathrm{m}}}{2}\alpha_{\mathrm{m}}^0\Re e[\hat{n}_{\mathrm{m}} \cdot \overleftrightarrow{G}_{\mathrm{res}} \cdot \hat{n}_{\mathrm{m}}] \tag{25}$$

$$\gamma_{\mathrm{m}} = \gamma_{\mathrm{m}}^0 + \alpha_{\mathrm{m}}^0\omega_{\mathrm{m}}Im\left[\hat{n}_{\mathrm{m}} \cdot \overleftrightarrow{G}_{\mathrm{mm}} \cdot \hat{n}_{\mathrm{m}}\right] = \gamma_{\mathrm{m}}^0 + F_{\mathrm{p}}\gamma_m^{\mathrm{s}} \tag{26}$$

$$\gamma_{\mathrm{m}}' = \gamma_{\mathrm{m}}^0 + \alpha_{\mathrm{m}}^0\omega_{\mathrm{m}}Im\left[\hat{n}_{\mathrm{m}} \cdot \overleftrightarrow{G}_{\mathrm{res}} \cdot \hat{n}_{\mathrm{m}}\right] = \gamma_{\mathrm{m}}^0 + F_{\mathrm{p}}^{\mathrm{res}}\gamma_{\mathrm{m}}^{\mathrm{s}} \tag{27}$$

where $\delta\omega_{\mathrm{L}}$ is designated as the Lamb shift or the change of the vibrational frequency due to the self-interaction with the surrounding plasmonic cavity, γ_{m}^0 is the intrinsic spontaneous decay rate of the molecular vibration, F_{p} and $F_{\mathrm{p}}^{\mathrm{res}}$ are the dipolar and residual Purcell factors, and the primed variables

$\delta\omega_{\mathrm{L}}'$ and γ_{m}' are the corresponding residual Lamb shift and linewidth due to multipolar effects. Hence the plasmonic dipole can be rewritten as

$$\mu_{\mathrm{pl}} = \alpha_{\mathrm{p}} E_j \left[\frac{(\omega_{\mathrm{m}}+\delta\omega_{\mathrm{L}}')^2-\omega^2-i\omega\gamma_{\mathrm{m}}'}{(\omega_{\mathrm{m}}+\delta\omega_{\mathrm{L}})^2-\omega^2-i\omega\gamma_{\mathrm{m}}}\right] \tag{28}$$

This can be rewritten in terms of the total scattered power and in terms of a typical Fano lineshape:

$$P(\omega) = \frac{\omega^4|\alpha_{\mathrm{p}}E_j|^2}{12\pi\epsilon_0 c^3}|\mu_{\mathrm{pl}}|^2 \tag{29}$$

$$P(\omega) = \frac{\omega^4|\alpha_{\mathrm{p}}E_j|^2}{12\pi\epsilon_0 c^3}\cdot\frac{\left[\frac{\omega^2-(\omega_{\mathrm{m}}+\delta\omega_{\mathrm{L}})^2}{\omega\gamma_{\mathrm{m}}}+\frac{2(\delta\omega_{\mathrm{L}}-\delta\omega_{\mathrm{L}}')}{\gamma_{\mathrm{m}}}\right]^2+\left(\frac{\gamma_{\mathrm{m}}'}{\gamma_{\mathrm{m}}}\right)^2}{\left[\frac{\omega^2-(\omega_{\mathrm{m}}+\delta\omega_{\mathrm{L}})^2}{\omega\gamma_{\mathrm{m}}}\right]^2+1}$$

or (as in the main text),

$$P(\omega) = A\frac{(\nu+q)^2+B}{\nu^2+1} \tag{30}$$

where we can then identify each term as:

$$A = \frac{\omega^4|\alpha_{\mathrm{p}}E_j|^2}{12\pi\epsilon_0 c^3}, \qquad B = \left(\frac{\gamma_{\mathrm{m}}'}{\gamma_{\mathrm{m}}}\right)^2, \qquad \nu = \frac{\omega^2-(\omega_{\mathrm{m}}+\delta\omega_{\mathrm{L}})^2}{\omega\gamma_{\mathrm{m}}}, \quad \text{and the Fano } q = \frac{2(\delta\omega_{\mathrm{L}}-\delta\omega_{\mathrm{L}}')}{\gamma_{\mathrm{m}}}$$

With the additional residual self-interaction terms beyond the dipole approximation, the model can now accurately account for the asymmetric lineshape and the increased extinction of the coupled molecular vibration, as seen in Figure S18a. From the fitted models, we can extract the molecular Lamb shift (always to lower energy), which can be as large as 60% of the molecular vibrational linewidth (Figure S18b).

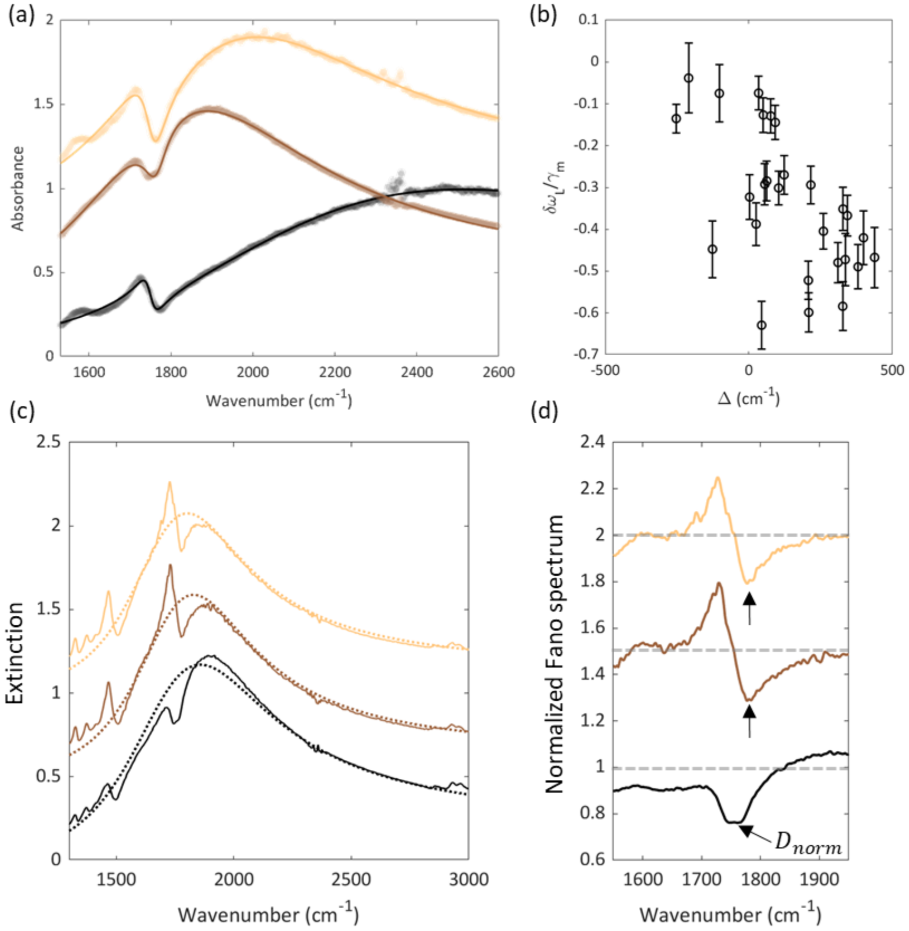

Figure S18. (a) Modified Fano fits of SEIRA spectra at various detuning of plasmonic mode from molecular vibration, showing the quality of the fit to the measured spectra. (b) Extracted molecular Lamb shift normalized to the molecular vibrational linewidth as a function of detuning ($\boldsymbol{\Delta} = \boldsymbol{\omega}_{\mathbf{BPP}}$-$\boldsymbol{\omega}_{\mathbf{m}}$) of the molecular mode to the plasmonic mode. (c) Plasmon extinction (dotted line) over NP7ML spectra with detuning $\boldsymbol{\Delta} \sim \mathbf{0}$ (solid line) and (d) Normalized Fano spectra obtained by dividing solid line spectra in (c) with dotted line plasmon extinction. Arrows mark position where $\boldsymbol{D}_{\mathbf{norm}}$ is evaluated at the location of $\boldsymbol{\omega}_{\mathbf{dip}}$

The dip strength D_{norm} can be measured by fitting the plasmonic resonance background and dividing the absorption spectrum by this resonance background (Figure S18c). For each measured spectrum, the dip magnitude D_{norm} at the molecular resonance (ω_{m}=1732cm^{-1}) is extracted from the normalized spectrum (Figure S18d), which is then used to determine the optomechanical coupling strength g. The normalized dip magnitude D_{norm} at the molecular resonance (ω_{m}=1732cm^{-1}) can be expressed from Eqn (28) as:

$$D_{\mathrm{norm}} \approx 1 - \left|\frac{\mu_{\mathrm{pl}}(\omega=\omega_{\mathrm{dip}})}{\mu_{\mathrm{pl}}^{0}(\omega=\omega_{\mathrm{dip}})}\right|^{2} \qquad (31)$$

where $\mu_{\rm pl}^{0}(\omega = \omega_{\rm dip})$ is the plasmonic dipole moment in the absence of the molecular vibration. From the modified Fano equation (29) above, we can then relate $D_{\rm norm} \simeq 1 - B = 1 - \left|\frac{\gamma_{\rm m}'}{\gamma_{\rm m}}\right|^{2}$ and hence we can obtain the coupling strength as a function of $D_{\rm norm}$:

$$g \simeq \left[\frac{\gamma_{\rm p}\gamma_{\rm m}'}{4}\left(\sqrt{\frac{1}{1-D_{\rm norm}}} - 1\right)\right]^{\frac{1}{2}} \quad (32)$$

To analyse the impact of the multipolar effects on the molecular self interaction $\overleftrightarrow{G}_{\rm res}$, we consider the generalized Fano asymmetry parameter $q = \frac{2(\delta\omega_{\rm L} - \delta\omega_{\rm L}')}{\gamma_{\rm m}}$ and substitute the expressions for $\delta\omega_{\rm L} = -\frac{\omega_{\rm m}}{2}\alpha_{\rm m}^{0}\Re e[\hat{n}_{\rm m}\cdot\overleftrightarrow{G}_{\rm mm}\cdot\hat{n}_{\rm m}]$ and $\delta\omega_{\rm L}' = -\frac{\omega_{\rm m}}{2}\alpha_{\rm m}^{0}\Re e[\hat{n}_{\rm m}\cdot\overleftrightarrow{G}_{\rm res}\cdot\hat{n}_{\rm m}]$. As $\overleftrightarrow{G}_{\rm mm} = \overleftrightarrow{G}_{\rm mp}\alpha_{\rm p}\overleftrightarrow{G}_{\rm pm}$ and the field enhancement $\overleftrightarrow{M} = \overleftrightarrow{G}_{\rm mp}\alpha_{\rm p}$, we can assume a modified Lorentzian lineshape for the field enhancement term as $\overleftrightarrow{G}_{\rm pm}\overleftrightarrow{M} = G_{\rm pm}^{0}M_{0}\omega_{\rm p}^{2}/(\omega_{\rm p}^{2} - \omega^{2} - i\omega\gamma_{\rm p})$, where M_0 is in general a complex number. This yields under first order Taylor expansion a Fano factor

$$q \simeq -\frac{2}{\gamma_{\rm m}}\left(\frac{\omega_{\rm p}}{\gamma_{\rm p}}\right)^{2}\alpha_{\rm m}^{0}\,{\rm Re}\left[G_{\rm pm}^{0}M_{\rm o}\left(\nu + \frac{i\gamma_{\rm p}}{2}\right)\right] \quad (33)$$

This should be compared to the expression for a simple dipolar interaction, where:

$$q_{\rm dipole} \simeq -\frac{2}{\gamma_{\rm m}}\left(\frac{\omega_{\rm p}}{\gamma_{\rm p}}\right)^{2}\alpha_{\rm m}^{0}\alpha_{\rm p}^{0}\overleftrightarrow{G}_{\rm mp}\overleftrightarrow{G}_{\rm pm}\nu \quad (34)$$

In the pure dipolar interaction, at zero detuning ($\Delta = \nu = 0$) the Fano $q_{\rm dipole}$ is also zero and the dip in the SEIRA spectrum is symmetric. However, in the generalized case, $G_{\rm pm}^{0}M_{\rm o} \in \mathbb{C}$ and hence q need not be zero at zero detuning. This can be seen in the spectra in Figure S19d.

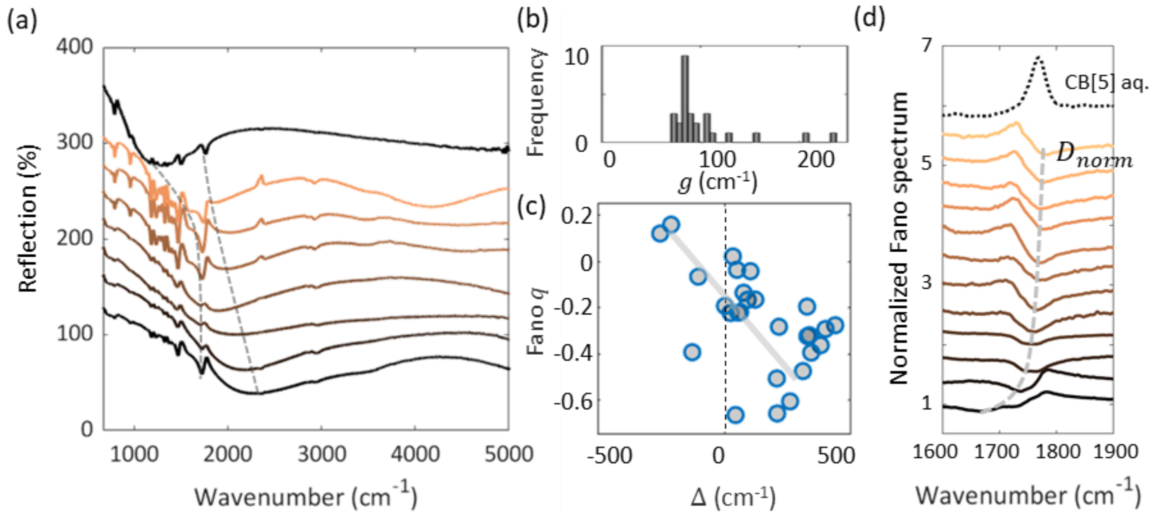

Figure S19. SEIRA spectra at different detunings between molecular resonance and plasmonic mode. (a) Reflection spectrum of 7ML to 8ML samples, with dotted grey line showing the two coupled plasmon-molecular vibration modes. (b) Extracted plasmon-vibration coupling strength by individually fitting modified Fano model to spectra in Fig. S18a. (c) Fano asymmetry parameter $\boldsymbol{q}$ as a function of plasmon-molecular vibration detuning $\boldsymbol{\Delta} = \boldsymbol{\omega_{\mathrm{BPP}}}$-$\boldsymbol{\omega_m}$ between molecular ($\boldsymbol{\omega_m}$) and plasmon ($\boldsymbol{\omega_{\mathrm{BPP}}}$) resonances (grey lines are guides to eye) to spectra in Fig. S18a. (d) Normalized Fano dip strength spectra, with dotted grey line marking Lamb shift and location of $\boldsymbol{D_{norm}}$ of the molecular resonance dip compared to the solution CB[5] spectrum (dashed black line). All spectra shifted for clarity.

Section S9: Radiative decay rate of CB[5] within cavity, Purcell factor, and mode volumes

We can then define an effective radiative Purcell factor (F_p) using the modified coupled oscillator model above, as the ratio of the modified damping/decay rates to the radiative spontaneous decay rate (γ_m^s):

$$F_p = \frac{\gamma_\mathrm{m} - \gamma'_\mathrm{m}}{\gamma_\mathrm{m}^\mathrm{s}} = \frac{4g^2}{\gamma_\mathrm{pl}\gamma_\mathrm{m}^\mathrm{s}} \qquad (35)$$

The spontaneous radiative decay rate in the infrared ($\gamma_\mathrm{m}^\mathrm{s}$) can be found from the Einstein decay coefficient [62], which can be measured from the liquid phase extinction coefficient of aqueous CB[5]. In IUPAC practical units [63], the decay coefficient is then:

$$\gamma_\mathrm{m}^\mathrm{s} = 2.88 \times 10^{-9} \cdot \omega_\mathrm{m}^2 \cdot A_\mathrm{p} \qquad (36)$$

where A_p is the area under the extinction spectrum corresponding to the vibrational mode ω_m. In Figure S20, this is the shaded area of the ω_m = 1765 cm^{-1} peak, and upon normalization by the 5mM concentration and 6µm path length of the liquid transmission cell (BaF_2 windows, Harrick Scientific), we obtain A_p = 3.72x10^{6} molL^{-1}cm^{-2}, and a radiative decay rate $\gamma_\mathrm{m}^\mathrm{s}$ = 33.5 kHz = 1.1x10^{-6} cm^{-1}. This is higher

than the expected decay rates of carbonyl and organic compounds in the gas phase [63] due to collisions with the surrounding water medium.

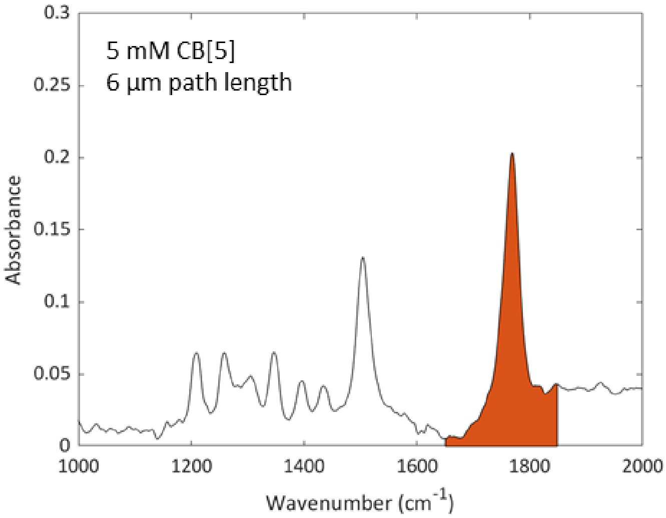

Figure S20. Absorbance spectrum of aqueous CB[5]. Shaded area represents integrated area for $\boldsymbol{\omega_m}$ = 1765 cm^{-1} C=O mode.

From the radiative decay rate and coupling strengths, we can calculate the average radiative Purcell factor as F_p = (5.5±0.9)x10^6 (Figure S21a). The Purcell factor at D_{norm}~0.4 and detuning Δ ~ 130 cm^{-1} (γ_m= 62 ± 5 cm^{-1} and γ'_m = 49 ± 4 cm^{-1}) is F_p = (11±0.9)x10^6. To validate this, we can independently estimate the Purcell factor using its conventional expression as a function of wavelength ($\lambda = 5.67\ \mu\mathrm{m} = 1769\ \mathrm{cm}^{-1}$) within a cavity of quality factor (Q), effective refractive index ($n_\mathrm{eff} \simeq 1.3$ chosen to compare to the spontaneous radiative decay rate of CB[5] in water) and mode volume (V):

$$F_\mathrm{p} = \frac{3}{4\pi^2}\left(\frac{\lambda}{n_\mathrm{eff}}\right)^3\left(\frac{Q}{V}\right) \qquad (37)$$

The expected mode volume from the equation above corresponding to F_p = (5.5±0.9)x10^6 is 2900 nm^3. By plotting the expected Purcell factor as a function of mode volume in Figure S21b, we can contextualize the Purcell factor obtained from the previous equation by the upper and lower bounds set by the sample nanostructure. The upper limit of F_p occurs when MIR light is confined to a single nanogap between two AuNPs (diameter 100 nm and facet diameter ~ radius/2 = 25 nm) giving a mode volume of 441 nm^3 and $F_\mathrm{p}{\sim}3\times10^7$. The lower limit of F_p is defined by the lateral coherence length of the plasmon [64] $d_\mathrm{pl} = (2\ln2/\pi)(\lambda^2/n_\mathrm{eff}\Delta\lambda)$ where $\Delta\lambda$ is the linewidth of the plasmon mode, and the thickness of the film (L~700 nm for 7 monolayers) to give mode volume $V \simeq \pi d_\mathrm{pl}^2 L$ = 8x10^8 nm^3 and F_p~19. The NP7ML films with a mode volume of 2900 nm^3 correspond to light being localized within 7 AuNP gaps, which is approximately the number of gaps within a septamer structure, closely approaching the upper limit of field confinement possible within this nanostructure, and thus explaining the high SEIRA enhancement factors. Within 2900 nm^3 or 7 gaps, there are approximately $N = 6\times10^{17}\ m^{-2}\cdot\pi\cdot(25\times10^{-9}\ m^2)^2 = 1178$ molecules. This results in a single molecule optomechanical coupling $g_0 = g/\sqrt{N}$ of 2.24 cm^{-1} = 0.3 meV.

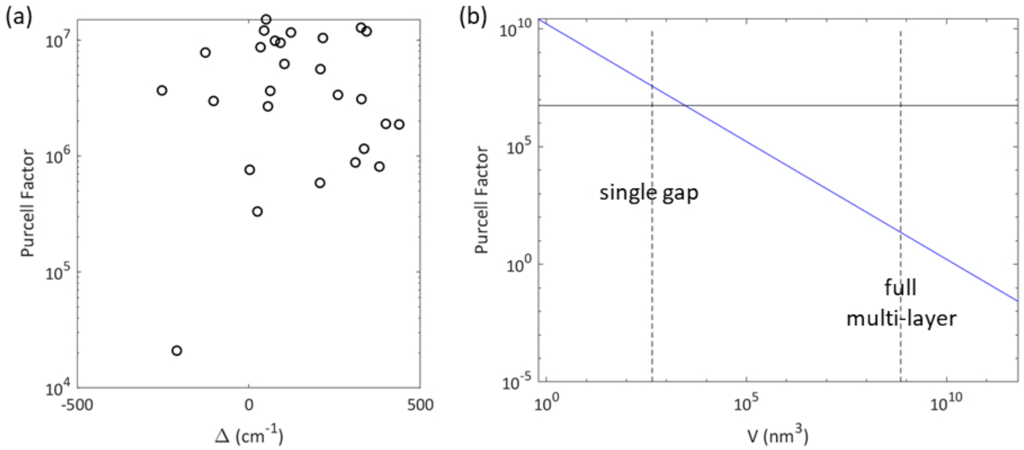

Figure S21. Purcell factor estimation. (a) Purcell factors calculated as a function of detuning. (b) Purcell Factor as a function of mode volume (blue line), estimated Purcell factor from decay rates (black horizontal line), and upper limit to Purcell factor from a single gap mode volume and lower limit from plasmon coherence length over entire multi-layer microcavity (vertical dashed lines).

Section S10: SEIRA spectra of decane-1-thiol monolayers on multilayer films

Samples of NP7ML films were soaked overnight in decane-1-thiol solution and subsequently rinsed in ethanol and dried in nitrogen gas flow. The IR reflectance spectra were then obtained and the spectrum of the bare film without decanethiol subtracted from it and background corrected with a 3rd order polynomial to remove the plasmonic resonance background. The resulting spectra in Fig.S21 show the spectrum of adsorbed 1-decanethiol, which can be compared to the spectrum of liquid 1-decanethiol, and showing peaks consistent with the literature [65].

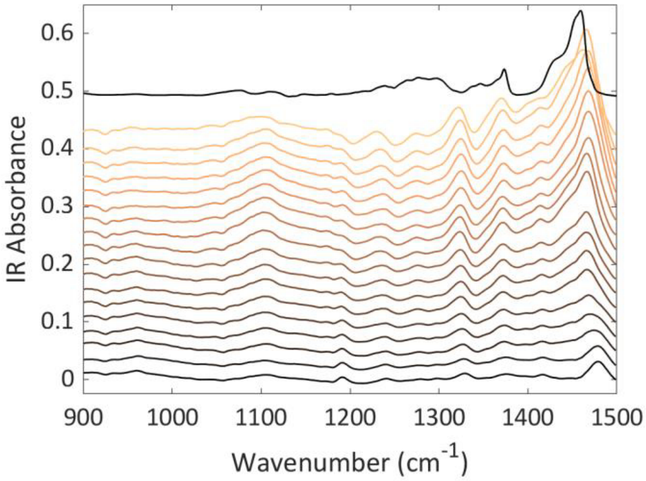

Figure S22. Background-corrected FTIR absorbance spectrum of decane-1-thiol on NP7ML films for different detunings of the plasmon mode, with black line indicating the spectrum of dodecane-1-thiol liquid from Ref.[65].

Section S11: Multi-particle Mie-scattering simulations of disorder in 2D platelets of AuNPs

The scattering spectra of 100 nm AuNPs were simulated using a multiple sphere Mie scattering code [66], for 2D arrays of AuNPs in a triangular lattice, with a gap size of 1.0 nm between AuNPs. The scattering spectrum redshifts from a septamer to larger platelets (Figure S23a), due to the presence of a collective superradiant plasmon mode extending across the platelet. The septamer has two main modes shown schematically in Figure S23b: a superradiant mode [67] with a superposition of dipoles oriented in the same direction for all 7 nanoparticles, and a sub-radiant mode where the centre nanoparticle dipole is oriented in the opposite direction [68]. In the far-field, the electric field of the sub-radiant and the superradiant mode interfere destructively, giving rise to the dip in intensity and a Fano lineshape due to the interference between the narrowband sub-radiant mode and the broadband superradiant mode. With each added generation of nanoparticles encircling the septamer, an additional dark mode develops with interference from dipoles encircling the inner layers aligned with or against the centre nanoparticle. These dark modes redshift as the platelet size becomes larger, and interfere with the superradiant mode that is becoming broader.

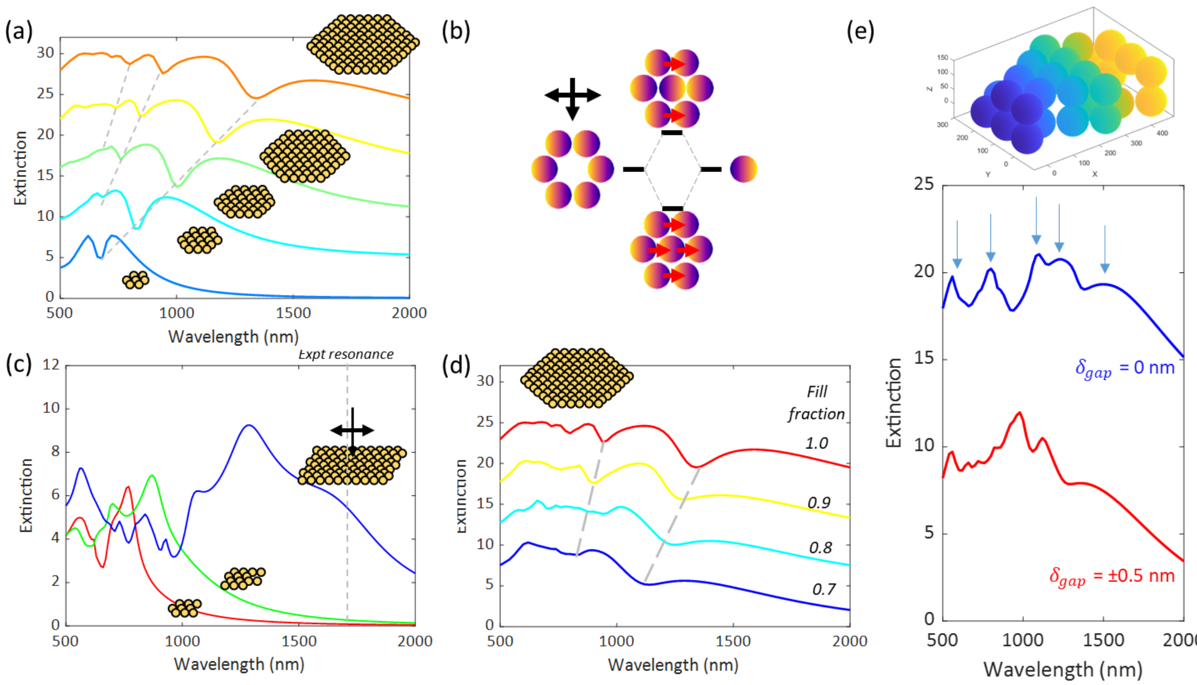

Figure S23. Triangular lattice platelets of 100 nm AuNPs with gap separation of 0.9 nm (a) Platelets of increasing size, by adding successive generations of rings to the middle septamer. Dotted lines show the dispersion of the Fano resonances. (b) Fano resonance due to hybridization of the hexamer mode with the internal NP dipole resulting in a dark and bright mode. Arrow indicate direction of propagation and polarization of incident light. Shading indicates charge distribution and plasmonic dipole direction on nanoparticles. (c) Symmetry-broken triangular platelets of size 4x3, 5x4 and 10x9 NPs. (d) Averaged extinction spectrum of hexagon AuNP platelet shown with different fill fractions/vacancies. (e) Averaged extinction spectrum of platelet depicted with random gap size disorder for fill fraction=0.7 with no gap size disorder δ_{gap}=0nm (blue) and δ_{gap}=±0.5 nm gap size disorder (red). Resonances indicated by arrows. Angle of incidence of light for all simulations is 90° to the surface normal of the platelet.

As the six-fold symmetry is broken in Figure S23c, the modes in the centre of the spectrum from 800 nm to 1300 nm become suppressed, as the sub-radiant modes become more numerous and complex, thus interfering destructively over a larger portion of the spectrum. If we introduce defects in the form of vacancies in the sixfold platelets however, we instead see a blueshift and broadening of the resonance with decreasing fill fraction in addition to the decrease in extinction at around 1000 nm. This is shown in Figure S23d where ten different random structures with a certain number of vacancy defects were generated and their averaged spectrum displayed. This is due to an effective decrease in the contiguous nanoparticle chain lengths in the structure. An unusual feature is the persistence of the Fano resonances, which in many previous studies have been claimed to be highly sensitive to disorder [69]. Instead, we see that the Fano resonances merely blueshift, and are highly robust to disorder when the gap sizes remain constant, even up to 30% vacancies.

We can introduce gap-size disorder by sampling the gap size from a normal distribution with width 0.5 nm centered at zero to model the deviation from the initial perfect 1 nm gap sizes defined by cucurbit-5-uril. Gap size disorder acts to blueshift and broaden the Fano resonance dips, destroying the previously sharp resonances below 1000 nm (Figure S23e blue arrows). A superposition of all the gap-size induced shift spectra thus blurs out the resonance and increases the width of the plasmon resonance.

Section S12: Repeatability of fabrication of NPnML films

Repeatable fabrication of multilayer NPnML films is shown below for independently prepared batches.

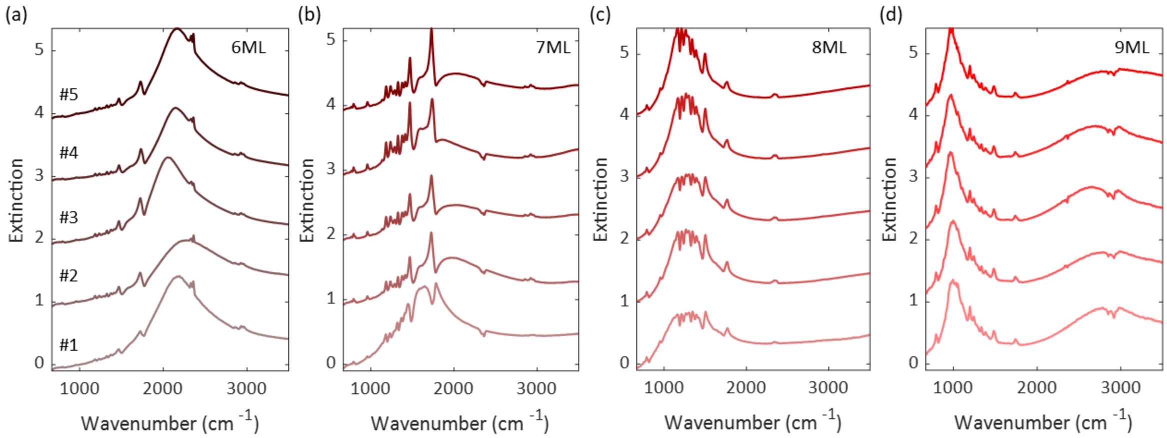

Figure S24. Repeatability of fabrication of NPnML films give reproducible IR extinction spectra (in absorbance units) for (a) n=6ML, (b) 7ML, (c) 8ML, (d) 9ML. Spectra offset for clarity. Spectra for each layer number are measured on different samples, prepared in independent batches.

Section S13: Flow sensing within NPnML films and reusability studies

A flow cell with CaF_2 windows is assembled on a laser machined silicon chip. A 3mm x 3mm square microfluidic chamber with 100 µm depth is written and connected to inlet and outlet glass capillaries of 40 µm inner diameter. The cell is then wet with isopropyl alcohol (IPA) and connected to a syringe pump. LP57 battery electrolyte (EC/EMC 3:7 (v) +1M $LiPF_6$) is then pumped in at 0.5 µL min^{-1}, preserving the capillary bridge between inlet and outlet capillaries through the NPnML film. FTIR spectra are recorded over time on an FTIR microscope (microscope as described in Methods section) at the centre of the microfluidic chamber. The FTIR time course was collected with 10 scans, over a 100µm x 100µm spot size, and with a resolution of 8 cm^{-1} every 30s.

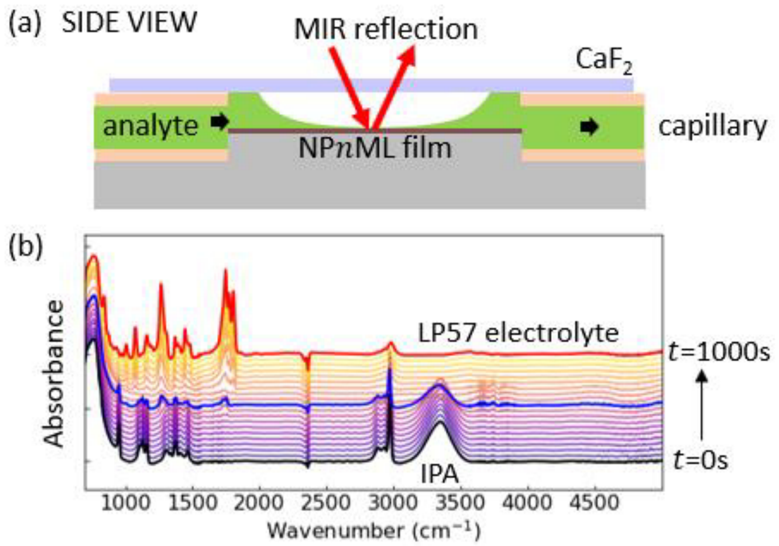

Figure S25. Optically-accessible flow cell for flow IR reflectance sensing. (a) Schematic with flow inlet and outlet, substrate coated with NPnML film, and IR-transparent CaF_2 top window. (b) FTIR reflectance spectra with time (in absorbance units, plasmonic background subtracted), showing flow replacement from isopropanol to a battery electrolyte (LP57).

Reusability of the substrates is shown in Figure S26 below, which shows the ability to oxygen-plasma clean the substrate surface and reintroduce an analyte (isopropanol). The oxygen plasma treatment is carried out for 60 min subsequent to layer deposition (oxygen mass flow of 30 sccm at 90 % RF power, Diener electronic GmbH + Co. KG).

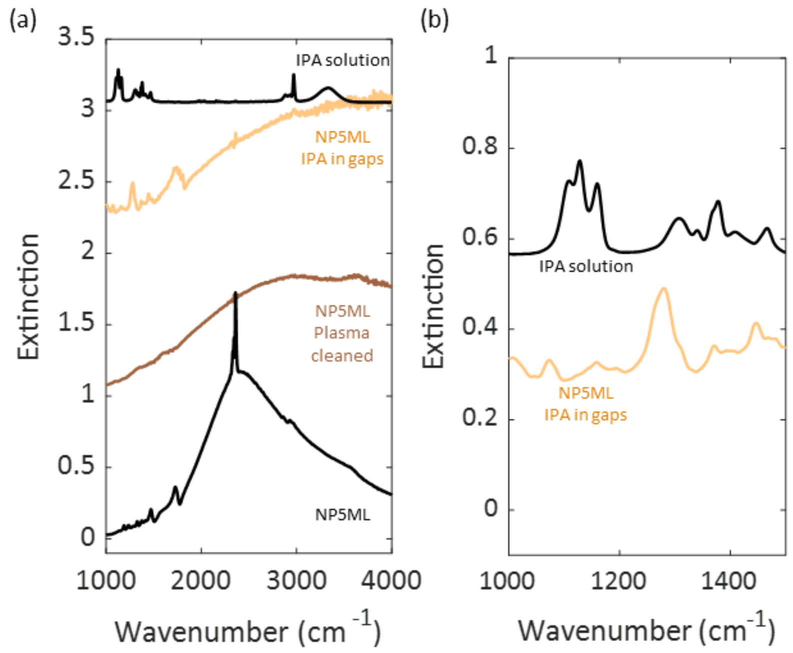

Figure S26. Reusability of NPnML films showing (a) native NP5ML spectra, NP5ML after oxygen plasma cleaning, NP5ML after oxygen plasma cleaning and flow of thin isopropanol (IPA) layer, compared to spectra of pure IPA solution. (b) Expanded spectrum of the IPA bands adsorbed on gold and within solution.

REFERENCES

1. Taylor, R. W.; Lee, T.-C.; Scherman, O. A.; Esteban, R.; Aizpurua, J.; Huang, F. M.; Baumberg, J. J.; Mahajan, S., Precise subnanometer plasmonic junctions for SERS within gold nanoparticle assemblies using cucurbit [n] uril “glue”. *ACS Nano* **2011,** *5* (5), 3878-3887.
2. De Nijs, B.; Bowman, R. W.; Herrmann, L. O.; Benz, F.; Barrow, S. J.; Mertens, J.; Sigle, D. O.; Chikkaraddy, R.; Eiden, A.; Ferrari, A., Unfolding the contents of sub-nm plasmonic gaps using normalising plasmon resonance spectroscopy. *Faraday Discussions* **2015,** *178*, 185-193.
3. Thomas, R.; Thomas, A.; Pullanchery, S.; Joseph, L.; Somasundaran, S. M.; Swathi, R. S.; Gray, S. K.; Thomas, K. G., Plexcitons: the role of oscillator strengths and spectral widths in determining strong coupling. *ACS Nano* **2018,** *12* (1), 402-415.
4. Mueller, N. S.; Pfitzner, E.; Okamura, Y.; Gordeev, G.; Kusch, P.; Lange, H.; Heberle, J.; Schulz, F.; Reich, S., Surface-Enhanced Raman Scattering and Surface-Enhanced Infrared Absorption by Plasmon Polaritons in Three-Dimensional Nanoparticle Supercrystals. *ACS Nano* **2021,** *15* (3), 5523-5533.
5. Johnson, P. B.; Christy, R.-W., Optical constants of the noble metals. *Physical Review B* **1972,** *6* (12), 4370.
6. Sönnichsen, C.; Franzl, T.; Wilk, T.; von Plessen, G.; Feldmann, J.; Wilson, O.; Mulvaney, P., Drastic reduction of plasmon damping in gold nanorods. *Physical Review Letters* **2002,** *88* (7), 077402.
7. Benz, F.; de Nijs, B.; Tserkezis, C.; Chikkaraddy, R.; Sigle, D. O.; Pukenas, L.; Evans, S. D.; Aizpurua, J.; Baumberg, J. J., Generalized circuit model for coupled plasmonic systems. *Optics Express* **2015,** *23* (26), 33255-33269.
8. Olmon, R. L.; Slovick, B.; Johnson, T. W.; Shelton, D.; Oh, S.-H.; Boreman, G. D.; Raschke, M. B., Optical dielectric function of gold. *Physical Review B* **2012,** *86* (23), 235147.
9. Mahajan, S.; Lee, T.-C.; Biedermann, F.; Hugall, J. T.; Baumberg, J. J.; Scherman, O. A., Raman and SERS spectroscopy of cucurbit [n] urils. *Physical Chemistry Chemical Physics* **2010,** *12* (35), 10429-10433.
10. Grishaeva, T. N.; Masliy, A. N.; Kuznetsov, A. M., Water structuring inside the cavities of cucurbit [n] urils (n= 5–8): a quantum-chemical forecast. *Journal of Inclusion Phenomena and Macrocyclic Chemistry* **2017,** *89* (3-4), 299-313.
11. Seah, M. P.; Dench, W., Quantitative electron spectroscopy of surfaces: A standard data base for electron inelastic mean free paths in solids. *Surface and interface analysis* **1979,** *1* (1), 2-11.
12. Liu, Y.; Ozolins, V., Self-assembled monolayers on Au (111): structure, energetics, and mechanism of reconstruction lifting. *The Journal of Physical Chemistry C* **2012,** *116* (7), 4738-4747.
13. Sotthewes, K.; Kap, O. z.; Wu, H.; Thompson, D.; Huskens, J.; Zandvliet, H. J., Ordering of air-oxidized decanethiols on Au (111). *The Journal of Physical Chemistry C* **2018,** *122* (15), 8430-8436.
14. Matei, D.; Muzik, H.; Gölzhäuser, A.; Turchanin, A., Structural investigation of 1, 1′ -biphenyl-4-thiol self-assembled monolayers on Au (111) by scanning tunneling microscopy and low-energy electron diffraction. *Langmuir* **2012,** *28* (39), 13905-13911.
15. Hwang, I.; Kim, M.; Yu, J.; Lee, J.; Choi, J. H.; Park, S. A.; Chang, W. S.; Lee, J.; Jung, J. Y., Ultrasensitive molecule detection based on infrared metamaterial absorber with vertical nanogap. *Small Methods* **2021,** *5* (8), 2100277.
16. Wang, D.; Wang, X.; Lin, H.; Wang, B.; Jiang, J.; Li, Z., Surface-Enhanced Infrared Absorption of Ligands on Colloidal Gold Nanowires through Resonant Coupling. *Analytical Chemistry* **2020,** *92* (5), 3494-3498.
17. Wang, H.; Kundu, J.; Halas, N. J., Plasmonic nanoshell arrays combine surface‐enhanced vibrational spectroscopies on a single substrate. *Angewandte Chemie International Edition* **2007,** *46* (47), 9040-9044.

18. Le, F.; Brandl, D. W.; Urzhumov, Y. A.; Wang, H.; Kundu, J.; Halas, N. J.; Aizpurua, J.; Nordlander, P., Metallic nanoparticle arrays: a common substrate for both surface-enhanced Raman scattering and surface-enhanced infrared absorption. *ACS Nano* **2008,** *2* (4), 707-718.
19. Kundu, J.; Le, F.; Nordlander, P.; Halas, N. J., Surface enhanced infrared absorption (SEIRA) spectroscopy on nanoshell aggregate substrates. *Chemical Physics Letters* **2008,** *452* (1-3), 115-119.
20. Yin, H.; Li, N.; Si, Y.; Zhang, H.; Yang, B.; Wang, J., Gold nanonails for surface-enhanced infrared absorption. *Nanoscale Horizons* **2020,** *5* (8), 1200-1212.
21. Li, N.; Yin, H.; Zhuo, X.; Yang, B.; Zhu, X. M.; Wang, J., Infrared ‐ Responsive Colloidal Silver Nanorods for Surface ‐ Enhanced Infrared Absorption. *Advanced Optical Materials* **2018,** *6* (17), 1800436.
22. Stanglmair, C.; Neubrech, F.; Pacholski, C., Chemical Routes to Surface Enhanced Infrared Absorption (SEIRA) Substrates. *Zeitschrift für Physikalische Chemie* **2018,** *232* (9-11), 1527-1539.
23. Nong, J.; Tang, L.; Lan, G.; Luo, P.; Li, Z.; Huang, D.; Shen, J.; Wei, W., Combined Visible Plasmons of Ag Nanoparticles and Infrared Plasmons of Graphene Nanoribbons for High ‐ Performance Surface ‐ Enhanced Raman and Infrared Spectroscopies. *Small* **2021,** *17* (1), 2004640.
24. Henry, W. A.; Biris, A. S.; Watanabe, F.; Huntington, T. E.; Owen, A. R.; Price, A. S.; Golden, J. W.; Primm, K. M.; Wilde, B. M.; Sweere, M. D., Surface-enhanced infrared absorption studies of copper nanostructures formed by oblique-angle deposition. *Chemical Physics Letters* **2016,** *663*, 111-114.
25. Seiça, A. F.; Iqbal, M. H.; Carvalho, A.; Choe, J.-y.; Boulmedais, F.; Hellwig, P., Study of Membrane Protein Monolayers Using Surface-Enhanced Infrared Absorption Spectroscopy (SEIRAS): Critical Dependence of Nanostructured Gold Surface Morphology. *ACS Sensors* **2021,** *6* (8), 2875-2882.
26. Bibikova, O.; Haas, J.; López-Lorente, A. I.; Popov, A.; Kinnunen, M.; Meglinski, I.; Mizaikoff, B., Towards enhanced optical sensor performance: SEIRA and SERS with plasmonic nanostars. *Analyst* **2017,** *142* (6), 951-958.
27. Srajer, J.; Schwaighofer, A.; Ramer, G.; Rotter, S.; Guenay, B.; Kriegner, A.; Knoll, W.; Lendl, B.; Nowak, C., Double-layered nanoparticle stacks for surface enhanced infrared absorption spectroscopy. *Nanoscale* **2014,** *6* (1), 127-131.
28. Huck, C.; Neubrech, F.; Vogt, J.; Toma, A.; Gerbert, D.; Katzmann, J.; Härtling, T.; Pucci, A., Surface-enhanced infrared spectroscopy using nanometer-sized gaps. *ACS Nano* **2014,** *8* (5), 4908-4914.
29. Aouani, H.; Sipova, H.; Rahmani, M.; Navarro-Cia, M.; Hegnerova, K.; Homola, J.; Hong, M.; Maier, S. A., Ultrasensitive broadband probing of molecular vibrational modes with multifrequency optical antennas. *ACS Nano* **2013,** *7* (1), 669-675.
30. Cheng, F.; Yang, X.; Gao, J., Ultrasensitive detection and characterization of molecules with infrared plasmonic metamaterials. *Scientific reports* **2015,** *5* (1), 1-10.
31. Neubrech, F.; Pucci, A.; Cornelius, T. W.; Karim, S.; García-Etxarri, A.; Aizpurua, J., Resonant plasmonic and vibrational coupling in a tailored nanoantenna for infrared detection. *Physical Review Letters* **2008,** *101* (15), 157403.
32. D'Andrea, C.; Bochterle, J.; Toma, A.; Huck, C.; Neubrech, F.; Messina, E.; Fazio, B.; Marago, O. M.; Di Fabrizio, E.; Lamy de La Chapelle, M., Optical nanoantennas for multiband surface-enhanced infrared and Raman spectroscopy. *ACS Nano* **2013,** *7* (4), 3522-3531.
33. Huck, C.; Vogt, J.; Sendner, M.; Hengstler, D.; Neubrech, F.; Pucci, A., Plasmonic enhancement of infrared vibrational signals: nanoslits versus nanorods. *ACS Photonics* **2015,** *2* (10), 1489-1497.
34. Bagheri, S.; Giessen, H.; Neubrech, F., Large ‐ Area Antenna ‐ Assisted SEIRA Substrates by Laser Interference Lithography. *Advanced Optical Materials* **2014,** *2* (11), 1050-1056.
35. Bochterle, J. r.; Neubrech, F.; Nagao, T.; Pucci, A., Angstrom-scale distance dependence of antenna-enhanced vibrational signals. *ACS Nano* **2012,** *6* (12), 10917-10923.

36. Pfitzner, E.; Seki, H.; Schlesinger, R.; Ataka, K.; Heberle, J., Disc antenna enhanced infrared spectroscopy: From self-assembled monolayers to membrane proteins. *ACS Sensors* **2018,** *3* (5), 984-991.
37. Vogt, J.; Huck, C.; Neubrech, F.; Toma, A.; Gerbert, D.; Pucci, A., Impact of the plasmonic near- and far-field resonance-energy shift on the enhancement of infrared vibrational signals. *Physical Chemistry Chemical Physics* **2015,** *17* (33), 21169-21175.
38. Bagheri, S.; Weber, K.; Gissibl, T.; Weiss, T.; Neubrech, F.; Giessen, H., Fabrication of square-centimeter plasmonic nanoantenna arrays by femtosecond direct laser writing lithography: effects of collective excitations on SEIRA enhancement. *ACS Photonics* **2015,** *2* (6), 779-786.
39. Adato, R.; Yanik, A. A.; Amsden, J. J.; Kaplan, D. L.; Omenetto, F. G.; Hong, M. K.; Erramilli, S.; Altug, H., Ultra-sensitive vibrational spectroscopy of protein monolayers with plasmonic nanoantenna arrays. *Proceedings of the National Academy of Sciences* **2009,** *106* (46), 19227-19232.
40. Yoo, D.; Mohr, D. A.; Vidal-Codina, F.; John-Herpin, A.; Jo, M.; Kim, S.; Matson, J.; Caldwell, J. D.; Jeon, H.; Nguyen, N.-C., High-contrast infrared absorption spectroscopy via mass-produced coaxial zero-mode resonators with sub-10 nm gaps. *Nano letters* **2018,** *18* (3), 1930-1936.
41. Weber, K.; Nesterov, M. L.; Weiss, T.; Scherer, M.; Hentschel, M.; Vogt, J.; Huck, C.; Li, W.; Dressel, M.; Giessen, H., Wavelength scaling in antenna-enhanced infrared spectroscopy: Toward the far-IR and THz region. *ACS Photonics* **2017,** *4* (1), 45-51.
42. Rodrigo, D.; Tittl, A.; Ait-Bouziad, N.; John-Herpin, A.; Limaj, O.; Kelly, C.; Yoo, D.; Wittenberg, N. J.; Oh, S.-H.; Lashuel, H. A., Resolving molecule-specific information in dynamic lipid membrane processes with multi-resonant infrared metasurfaces. *Nature Communications* **2018,** *9* (1), 1-9.
43. Etezadi, D.; Warner IV, J. B.; Lashuel, H. A.; Altug, H., Real-time in situ secondary structure analysis of protein monolayer with mid-infrared plasmonic nanoantennas. *ACS Sensors* **2018,** *3* (6), 1109-1117.
44. Brown, L. V.; Zhao, K.; King, N.; Sobhani, H.; Nordlander, P.; Halas, N. J., Surface-enhanced infrared absorption using individual cross antennas tailored to chemical moieties. *Journal of the American Chemical Society* **2013,** *135* (9), 3688-3695.
45. Cerjan, B.; Yang, X.; Nordlander, P.; Halas, N. J., Asymmetric aluminum antennas for self-calibrating surface-enhanced infrared absorption spectroscopy. *ACS Photonics* **2016,** *3* (3), 354-360.
46. Yue, W.; Kravets, V.; Pu, M.; Wang, C.; Zhao, Z.; Hu, Z., Multiple-resonant pad-rod nanoantennas for surface-enhanced infrared absorption spectroscopy. *Nanotechnology* **2019,** *30* (46), 465206.
47. Dregely, D.; Neubrech, F.; Duan, H.; Vogelgesang, R.; Giessen, H., Vibrational near-field mapping of planar and buried three-dimensional plasmonic nanostructures. *Nature Communications* **2013,** *4* (1), 1-9.
48. Li, Y.; Yan, H.; Farmer, D. B.; Meng, X.; Zhu, W.; Osgood, R. M.; Heinz, T. F.; Avouris, P., Graphene plasmon enhanced vibrational sensing of surface-adsorbed layers. *Nano letters* **2014,** *14* (3), 1573-1577.
49. Li, Z.; Zhu, Y.; Hao, Y.; Gao, M.; Lu, M.; Stein, A.; Park, A.-H. A.; Hone, J. C.; Lin, Q.; Yu, N., Hybrid metasurface-based mid-infrared biosensor for simultaneous quantification and identification of monolayer protein. *ACS Photonics* **2019,** *6* (2), 501-509.
50. Vogt, J.; Zimmermann, S. r.; Huck, C.; Tzschoppe, M.; Neubrech, F.; Fatikow, S.; Pucci, A., Chemical identification of individual fine dust particles with resonant plasmonic enhancement of nanoslits in the infrared. *ACS Photonics* **2017,** *4* (3), 560-566.
51. Braun, A.; Maier, S. A., Versatile direct laser writing lithography technique for surface enhanced infrared spectroscopy sensors. *ACS Sensors* **2016,** *1* (9), 1155-1162.

52. Chen, K.; Duy Dao, T.; Nagao, T., Tunable nanoantennas for surface enhanced infrared absorption spectroscopy by colloidal lithography and post-fabrication etching. *Scientific reports* **2017,** *7* (1), 1-8.
53. Chong, X.; Zhang, Y.; Li, E.; Kim, K.-J.; Ohodnicki, P. R.; Chang, C.-h.; Wang, A. X., Surface-Enhanced Infrared Absorption: Pushing the Frontier for On-Chip Gas Sensing. *ACS Sensors* **2018,** *3* (1), 230-238.
54. Zvagelsky, R.; Chubich, D.; Pisarenko, A.; Bedran, Z.; Zhukova, E., Plasmonic metasurfaces as surface-enhanced infrared absorption substrates for optoelectronics: AlQ3 thin-film study. *The Journal of Physical Chemistry C* **2021,** *125* (8), 4694-4703.
55. Dong, L.; Yang, X.; Zhang, C.; Cerjan, B.; Zhou, L.; Tseng, M. L.; Zhang, Y.; Alabastri, A.; Nordlander, P.; Halas, N. J., Nanogapped Au antennas for ultrasensitive surface-enhanced infrared absorption spectroscopy. *Nano letters* **2017,** *17* (9), 5768-5774.
56. Omeis, F.; Santos Seica, A. F.; Bernard, R.; Javahiraly, N.; Majjad, H.; Moss, D.; Hellwig, P., Following the chemical immobilization of membrane proteins on plasmonic nanoantennas using infrared spectroscopy. *ACS Sensors* **2020,** *5* (7), 2191-2197.
57. Miao, X.; Yan, L.; Wu, Y.; Liu, P. Q., High-sensitivity nanophotonic sensors with passive trapping of analyte molecules in hot spots. *Light: Science & Applications* **2021,** *10* (1), 1-11.
58. Rodrigo, D.; Limaj, O.; Janner, D.; Etezadi, D.; García de Abajo, F. J.; Pruneri, V.; Altug, H., Mid-infrared plasmonic biosensing with graphene. *Science* **2015,** *349* (6244), 165-168.
59. Abb, M.; Wang, Y.; Papasimakis, N.; De Groot, C.; Muskens, O. L., Surface-enhanced infrared spectroscopy using metal oxide plasmonic antenna arrays. *Nano letters* **2014,** *14* (1), 346-352.
60. Pelton, M.; Storm, S. D.; Leng, H., Strong coupling of emitters to single plasmonic nanoparticles: exciton-induced transparency and Rabi splitting. *Nanoscale* **2019,** *11* (31), 14540-14552.
61. Zhang, Y.; Meng, Q.-S.; Zhang, L.; Luo, Y.; Yu, Y.-J.; Yang, B.; Zhang, Y.; Esteban, R.; Aizpurua, J.; Luo, Y., Sub-nanometre control of the coherent interaction between a single molecule and a plasmonic nanocavity. *Nature Communications* **2017,** *8* (1), 1-7.
62. Dunbar, R. C., Infrared radiative cooling of gas ‐ phase ions. *Mass Spectrometry Reviews* **1992,** *11* (4), 309-339.
63. Dunbar, R., Infrared radiative decay in complex molecules. *Spectrochimica Acta Part A: Molecular Spectroscopy* **1975,** *31* (5-6), 797-800.
64. Akcay, C.; Parrein, P.; Rolland, J. P., Estimation of longitudinal resolution in optical coherence imaging. *Applied Optics* **2002,** *41* (25), 5256-5262.
65. S.Matsuyama, S. K., K.Tanabe and T.Tamura, Spectral Database for Organic Compounds (SDBSWeb). National Institute of Advanced Industrial Science and Technology: 2018.
66. Mackowski, D.; Mishchenko, M., A multiple sphere T-matrix Fortran code for use on parallel computer clusters. *Journal of Quantitative Spectroscopy and Radiative Transfer* **2011,** *112* (13), 2182-2192.
67. Choudhary, S.; De Leon, I.; Swiecicki, S.; Awan, K. M.; Schulz, S. A.; Upham, J.; Alam, M. Z.; Sipe, J.; Boyd, R. W., Weak superradiance in arrays of plasmonic nanoantennas. *Physical Review A* **2019,** *100* (4), 043814.
68. Mirin, N. A.; Bao, K.; Nordlander, P., Fano resonances in plasmonic nanoparticle aggregates. *The Journal of Physical Chemistry A* **2009,** *113* (16), 4028-4034.
69. Luk'yanchuk, B.; Zheludev, N. I.; Maier, S. A.; Halas, N. J.; Nordlander, P.; Giessen, H.; Chong, C. T., The Fano resonance in plasmonic nanostructures and metamaterials. *Nature Materials* **2010,** *9* (9), 707.
